# The air mycobiome is decoupled from the soil mycobiome in the California San Joaquin Valley

**DOI:** 10.1111/mec.16640

**Published:** 2022-08-25

**Authors:** Robert Wagner, Liliam Montoya, Cheng Gao, Jennifer R. Head, Justin Remais, John W. Taylor

**Affiliations:** ^1^ Department of Plant & Microbial Biology University of California Berkeley Berkeley California USA; ^2^ Institute of Microbiology, Chinese Academy of Sciences Beijing China; ^3^ Division of Epidemiology University of California Berkeley Berkeley California USA; ^4^ Division of Environmental Health Sciences University of California Berkeley Berkeley California USA

**Keywords:** air, *Coccidioides*, dispersal, fungi, mycobiome, soil

## Abstract

Dispersal is a key force in the assembly of fungal communities and the air is the dominant route of dispersal for most fungi. Understanding the dynamics of airborne fungi is important for determining their source and for helping to prevent fungal disease. This understanding is important in the San Joaquin Valley of California, which is home to 4.2 million people and where the airborne fungus *Coccidioides* is responsible for the most important fungal disease of otherwise healthy humans, coccidioidomycosis. The San Joaquin Valley is the most productive agricultural region in the United States, with the principal crops grown therein susceptible to fungal pathogens. Here, we characterize the fungal community in soil and air on undeveloped and agricultural land in the San Joaquin Valley using metabarcoding of the internal transcribed spacer 2 variable region of fungal rDNA. Using 1,002 individual samples, we report one of the most extensive studies of fungi sampled simultaneously from air and soil using modern sequencing techniques. We find that the air mycobiome in the San Joaquin Valley is distinct from the soil mycobiome, and that the assemblages of airborne fungi from sites as far apart as 160 km are far more similar to one another than to the fungal communities in nearby soils. Additionally, we present evidence that airborne fungi in the San Joaquin Valley are subject to dispersal limitation and cyclical intra‐annual patterns of community composition. Our findings are broadly applicable to understanding the dispersal of airborne fungi and the taxonomic structure of airborne fungal assemblages.

## 1 INTRODUCTION

It has long been known that the air harbours microorganisms following Eherenberg's discovery of “infusoria” in dust samples collected off the coast of Africa nearly 200 years ago (Ehrenberg, 1830). Subsequent research has provided the foundation for our understanding of airborne microbial dispersal (Darwin, 1846; Pasteur, 1860), a fundamental process in community assembly (Nemergut et al., 2013; Vellend, 2010). Of particular interest are fungi because they primarily disperse through the air (Magyar et al., 2016; Talbot, 1997), can travel vast distances (Brown & Hovmøller, 2002) and have many dormancy mechanisms at their disposal (Lennon & Jones, 2011; Locey, 2010) which confer protection from damaging environmental conditions during transport (Dijksterhuis, 2019; Wyatt et al., 2013). Fungi provide many important ecosystem services such as establishing mutually beneficial relationships with plant species, breaking down leaf and woody material, and cycling carbon and nitrogen in soils (Baldrian, 2017; Becquer et al., 2019; Lustenhouwer et al., 2020; Read & Perez‐Moreno, 2003; Stuart & Plett, 2020). Many fungal species, especially airborne fungi, are associated with diseases affecting humans, crops, and wild plants and animals (Fisher et al., 2012). Modern studies using high‐throughput sequencing to describe the outdoor air microbiome are rare, and those focusing on fungi (the air *myco*biome) rarer still, despite the outdoor air medium harbouring diverse, spatially and temporally variable fungal populations (Barberán et al., 2015; Fierer et al., 2008; Frohlich‐Nowoisky et al., 2009; Núñez et al., 2019; Woo et al., 2013).

Fungal disease in the San Joaquin Valley (SJV) in California, which is home to 4.2 million people (US Census Bureau, 2019), is illustrative of the need to better understand airborne fungal dispersal. The fungus *Coccidioides* is a virulent airborne respiratory pathogen that is endemic to the SJV (Dixon, 2001; Egeberg & Ely, 1956; Kollath, Miller, & Barker, 2019; Nguyen et al., 2013; Ophüls, 1905) and is responsible for nearly 200 deaths and $3.9 billion in costs per year in the United States (Centers for Disease Control and Prevention, 2018; Gorris et al., 2021; Huang et al., 2012). Found primarily in California and Arizona, it remains unclear on what environmental medium *Coccidioides* primarily grows, its source of nutrition (Barker et al., 2012; Emmons, 1942; Kollath, Teixeira, et al., 2019; Taylor & Barker, 2019), or its precise means and range of dispersal (de Perio et al., 2019; Nicas, 2018; Pappagianis & Einstein, 1978; Schneider et al., 1997; Wilken et al., 2015). Agriculturally, the SJV is the most productive region in the United States (Food & Agriculture (CDFA), 2018), and plant pathogenic and parasitic fungi are responsible for significant losses in many of the chief crops grown therein (Baumgartner et al., 2019; Baumgartner & Rizzo, 2002; Camiletti et al., 2022; Holland et al., 2021). Determining the source and dispersal characteristics of airborne fungi can aid in preventing or mitigating fungal disease in humans and crops as well as offering a better understanding of fungal community ecology.

In what is still the most extensive modern outdoor air mycobiome work, significant differences in the distribution of fungi in settled dust have been demonstrated between regions at the continental scale (Barberán et al., 2015) despite a documented capacity amongst airborne fungi for long‐distance transport (Brown & Hovmøller, 2002; Prospero et al., 2005). The sampling of settled dust by Barberán et al. (2015) was largely focused in population centres and samples were broadly collected across North America, each at a single time point, including one sample from the SJV. A further analysis of the same data that focused on plant pathogens illustrated an association between certain pathogenic fungal taxa and geographical regions of the United States (Dietzel et al., 2019). Few high‐throughput sequencing studies, however, have paired aerobiology investigations of outdoor fungal communities with simultaneous sampling of the substrates on which they grow. Abrego et al. (2018) showed that the air mycobiomes of two individual samples taken at a single site were more similar to the air mycobiomes in sites >100 km away than to the soil mycobiomes at the same site collected several years previously (Abrego et al., 2018; Mäkipää et al., 2017). Their 2020 follow‐up publication reported less variation in the air mycobiome than the soil mycobiome, at distances up to 20 km, and also across a shift in land use from natural to urban (Abrego et al., 2020). Similarly, Kivlin et al. (2014) showed that the air mycobiomes from five sites, stretching ~115 km from Irvine, California to the vicinity of Mt San Jacinto, California, did not differ from one another, changed little over a 17‐month sampling period and were distinct from the soil mycobiome (Kivlin et al., 2014). Recently, Schiro et al. (2022) found fungi in dust more closely resembles the soil fungal community nearby than airborne fungi from distant sites, though the dust collected in their study consisted of surface soil that had been suspended using a portable wind tunnel.

Here we report the most extensive community‐level, high‐throughput sequencing study of fungi collected simultaneously from air and soil to date. Many studies have explored the air mycobiome in recent years, though to our knowledge only four other works have investigated both air and soil using high‐throughput sequencing, each with an order of magnitude fewer samples than we present here (Table S1). With just over 1,000 samples, we characterized the fungal communities within soils and inferred the assemblage of airborne fungi above these soils via settled dust from both undeveloped land and actively cultivated agricultural land within the SJV in California. Our expectation that fungi in settled dust would most closely resemble the local soil fungal community was surprisingly shown to be false. Instead, assemblages of fungi in settled dust more closely resembled fungi in settled dust from distant sites rather than resembling soil communities collected beneath our dust samplers. Principally, our community‐level investigation allowed us to discern patterns of fungal dispersal between the soil and airborne mycobiomes in the SJV and generate hypotheses regarding fungal pathogens in the region. By sampling in the SJV, we complement previous work by focusing on a critically important yet overlooked region, and our extensive sampling and temporally explicit approach provides a more comprehensive assessment of regional airborne fungal dispersal than preceding studies. This information will further our understanding of fungal dispersal as well as facilitate connections between epidemiological data on fungal disease of crops and humans with fungal dispersal dynamics, allowing more robust predictions to be generated and prevention strategies employed.

## 2 METHODS

**FIGURE 1:**
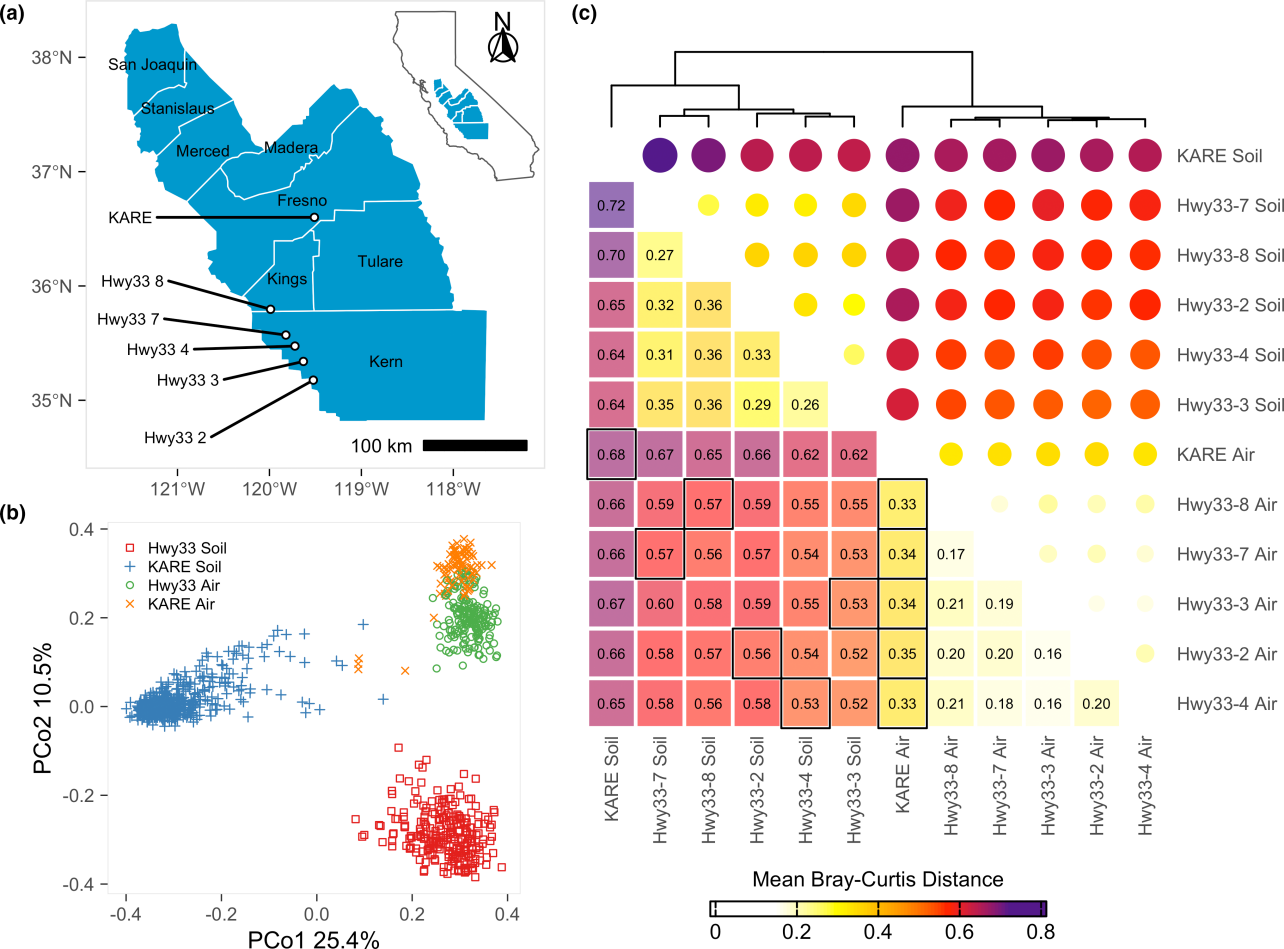
(a) Location of sampling sites on undeveloped land (Hwy33) and agricultural land (KARE) in the San Joaquin Valley with counties labelled. Inset shows the location of labelled counties within California. (b) Principal coordinate analysis of the Bray–Curtis dissimilarity between ITS2‐identified fungal species, demarcated by land use (Hwy33 vs. KARE) and sampling medium (soil vs. air), which separates into three distinct groups: agricultural (KARE) soil, wild (Hwy33) soil, and air from both agricultural and wild land (KARE *and* Hwy33). (c) Hierarchically clustered mean Bray–Curtis distances derived from principal coordinates between each pair of individual sites and sampling mediums. Distances were greatest between KARE and Hwy33 soils, and between soil and air samples, and least when comparing between only air samples. Air samples from both land uses were more similar to soils from Hwy33 than soils from KARE. Black boxes indicate comparisons between soil and air at the same site, and between air at KARE and air at each Hwy33 site.

Fungi in soil and in settled dust were sampled from undeveloped land (defined here as uncultivated and unirrigated land showing few signs of recent disturbance) adjacent to California highway 33 (Hwy33), at five sites spanning 80 km, monthly, from November 2017 to October 2018. These sites provide a north–south transect though one of the least developed areas in the SJV (Table S2). Hwy33 sites have aridic soils, receive between 15 and 25 cm of annual precipitation, and support a wild vegetation that includes *Nassella* spp., *Sporobolus* spp., *Suaeda nigra*, *Atriplex polycarpa* and *Adenostoma fasciculatum* (Griffith et al., 2016). Developed land along Hwy33 is primarily cropland, followed by pasture and sites of oil extraction interspersed with small urban areas (Griffith et al., 2016). Fungi in soil and in settled dust were also sampled from experimental sorghum fields at the Kearney Agricultural Research and Extension Center (KARE), 100 km northeast of the nearest Hwy33 site, in the summers of 2016, 2017 and 2018 (Gao et al., 2020). There is little undeveloped land near KARE (Griffith et al., 2016). At their farthest, these six sites extend across 160 km, a substantial portion of the SJV (Figure 1a; Table S2). Soils from agricultural land were collected as shallow soil cores from the upper organic soil layers (Gao et al., 2018, 2020), where both the highest density and a broad diversity of microbial and fungal species are encountered (Fierer et al., 2003; Hao et al., 2021). While soils from agricultural land were collected as shallow soil cores, we chose to collect soils on undeveloped land from within rodent burrows. Fungi generally inhabit places that are protected from stressors such as desiccation, high temperatures and UV irradiation in extreme environments (Makhalanyane et al., 2015; Santiago et al., 2018), and rodent burrows provide such a habitat and are nearly ubiquitous across the landscape in arid and semi‐arid ecosystems (Davidson & Lightfoot, 2008; Grinnell, 1923; Whitford & Kay, 1999). Our own experience confirmed this ubiquity in the SJV, with hundreds of burrows observed in the immediate vicinity at all Hwy33 sites. Rodent burrows have been shown to be rich in fungal diversity, owing not only to their environmental conditions but also to the nutrients provided by rodents and other macro‐organisms that reside in the burrows (Hawkins, 1996; Herrera et al., 1999; Miranda et al., 2019; Reichman et al., 1985). Given their unique characteristics when compared to the surrounding landscape, we expect that rodent burrows contribute greatly to soil fungal diversity in arid and semi‐arid regions.

### 2.1 Sampling and DNA extraction

At Hwy33 sites, soil was sampled from within rodent burrows using hemispherical collectors mounted on threaded rods inserted in the burrows as deeply as possible but no deeper than 30 cm. Ten burrows were sampled at each site in the first month (November 2017). Thereafter, due to time constraints, sampling was reduced to three burrows for all remaining months. Sampled burrows were not necessarily the same each month but were selected as close as possible to the coordinates sampled the previous month. There were no other selection criteria for burrows. Air was sampled by allowing dust to passively settle into empty, sterile, 10‐cm Petri dish bottoms placed 50 cm off the ground on a polyvinylchloride pipe and protected from precipitation beneath a plastic cone (Figure S1). Three passive dust collectors were placed in a triangular formation at each site with two collectors 5 m apart on an east–west axis (the western of which was at the coordinates of soil sampling) that lay 50 m north of the third collector. After 1 month of exposure, Petri dish bottoms were retrieved and covered with sterile lids for transport to the laboratory, and replaced with clean, sterile bottoms, a process that was repeated monthly at each Hwy33 site from November 2017 to October 2018. At KARE, soils were sampled by collecting soil cores 3 cm in diameter and 15 cm deep, as previously described (Gao et al., 2020), which spans soil depths that incorporate a range of agricultural soil fungal diversity (Schmidt et al., 2019). KARE samples were collected from May to September in 2016, June to October in 2017, and July to October in 2018, with total monthly replicates ranging from six to 90 soil samples. Air samples of passively settled dust were taken at KARE in September and October of 2017 (Gao et al., 2020) and from June to October of 2018 as described above with one difference; at KARE, 13 air samplers were arrayed at the corners of nested squares with sides of 10, 20, 40 and 80 m. Settled dust was retrieved from all Petri dish bottoms using sterile, DNA‐free swabs moistened in sterile, DNA‐free, distilled water. Swabs were cut from their wooden sticks, placed into buffer and disrupted by bead beating followed by DNA extraction using the MoBio Powersoil DNA kit (MoBio). Soil, 0.25 g, was added to buffer and DNA extracted using the same kit. DNA was quantified using a Qubit dsDNA HS Assay kit (Life Technologies) and then diluted to 5 ng μl^−1^.

### 2.2 PCR amplification and sequencing

For Hwy33 samples, the ITS2 region was PCR‐ (polymerase chain reaction) amplified from extracted DNA with the 5.8SFun (AACTTTYRRCAAYGGATCWCT) and ITS4Fun (AGCCTCCGCTTATTGATATGCTTAART) primers (Taylor et al., 2016) using the AccuStart II PCR SuperMix kit (Quantabio). The reaction mixture contained 2 μl of undiluted template DNA, 2.5 μl each of 50 μm forward and reverse primer, 12.5 μl AccuStart II PCR SuperMix, 2.5 μl of nuclease‐free water and 3 μl bovine serum albumin (BSA). A negative control consisted of 2 μl of nuclease‐free water in the place of template DNA. Amplification was performed on the Gene Amplification PCR System (Bio‐Rad Laboratories) under the following conditions: one cycle of 96°C for 2 min, 35 cycles of 94°C for 30 s, 58°C for 40 s and 72°C for 2 min, and one cycle of 72°C for 10 min. The PCR product was quantified using the Qubit dsDNA HS Assay kit (Life Technologies) and sent to the QB3 Vincent J. Coates Genomics Sequencing Laboratory (University of California, Berkeley, CA, USA), where samples were assigned unique dual indices to avoid barcode bleed/tag‐jumping (Carøe & Bohmann, 2020; Zinger et al., 2019), and sequenced on the MiSeq platform using the paired‐end PE300 chemistry (Illumina). For KARE samples, the molecular protocols were identical with the following exceptions: template DNA was diluted to 5 ng μl^−1^, BSA was not added and 5PRIME HotMaster Mix (Eppendorf‐5Prime), now discontinued, was used instead of the AccuStart II PCR SuperMix (Gao et al., 2020).

### 2.3 Sequence processing

All sequence processing was done in qiime 2 version 2019.10.0 (Bolyen et al., 2019) and sequence runs were visually inspected for quality using the *summarize* command. Sequences were denoised using the *denoise‐paired* command in dada2 (Callahan et al., 2016), and primer sequences were removed, paired‐end reads were joined, and bases trimmed at the beginning and end of every read once the median quality score dropped below 25. Unpaired reads (roughly 2% of reads) were discarded. A naïve Bayes classifier was trained with the UNITE database (UNITE Community, 2019) at 97% similarity using the *feature‐classifier fit‐classifier‐naive‐bayes* command, and operational taxonomic units (OTUs) were assigned with the *feature‐classifier classify‐sklearn* command (Bokulich et al., 2018; Pedregosa et al., 2011). “Unidentified” taxa could be matched to an unidentified UNITE database sequence entry, indicating that this sequence had been found in the environment, though the taxon associated with it remains unknown. “Unspecified” taxa, on the other hand, were algorithmically categorized by *classify‐sklearn*, a machine learning method, at a certain taxonomic level but were not matched to a specific UNITE database entry. All data and metadata supporting the findings of this study have been deposited in the NCBI Sequence Read Archive (www.ncbi.nlm.nih.gov/sra) with the following accession numbers: PRJNA736543 (Wagner, 2021a), PRJNA736167 (Wagner, 2021b) and PRJNA736519 (Wagner, 2021c). All code used to convert raw sequencing data (FASTQ files) into the taxonomic tables used in this study are included as Appendix S1.

### 2.4 Statistical analysis

Statistical analyses used R version 4.0.2 (R Core Team, 2020) and vegan version 2.5.6 (Oksanen et al., 2019). Taxa were analysed at the species level and those represented by only one DNA sequence amongst all samples were removed. Unidentified and unspecified taxa were also removed for all community‐level statistical analyses, but were kept for generation of taxonomic figures. Taxon tables were then transformed (square‐root‐transformed to reduce the effect of a few dominant taxa and Wisconsin double‐standardized) before calculating Bray–Curtis dissimilarity (Bray & Curtis, 1957; Legendre & Gallagher, 2001). Wisconsin double standardization first divides each taxon by the most abundant taxon across all samples, followed by division across all taxa for each sample to calculate proportional relative effect sizes. These transformations make taxa comparable across samples regardless of sample size. Taxa were not rarefied as rarefaction of microbiome data can introduce bias and needlessly throw out data (McMurdie & Holmes, 2014; Willis, 2019). We found no effect on our findings in tests of rarefaction or the inclusion of sequencing depth as a covariate (Weiss et al., 2017).

Differences in the fungal community between factors (land use, site, year, month and sampling medium) were assessed using a nested PERMANOVA (Anderson, 2001) on Bray–Curtis dissimilarities with the adonis2 function. Bray–Curtis dissimilarities were visualized using principal coordinate analysis (PCoA) with ape version 5.6.2 (Paradis & Schliep, 2019) and clustering of principal coordinate scores used ward.D2 distances (Murtagh & Legendre, 2014). Permutations (1,000) were left unstratified as stratifying (block permutations) did not change the nested PERMANOVA results. Pairwise differences between land use and sampling medium were assessed using pairwiseadonis (Arbizu, 2019) with 1,000 permutations. Significance of the relationship between temporal and geographical distance (distance–decay), and Bray–Curtis dissimilarity, was correlated (Pearson) using the Mantel test (Legendre & Legendre, 2012; Mantel & Valand, 1970) with unstratified permutations (1,000). Significant differences between factors in the strength (slope) of the distance–decay relationship were established when a significant interaction was present using linear regression. A linear mixed effects model was used to test for differences in *Onygenales* abundance between land use and sampling medium combinations as fixed effects, and month as a random effect, using lme4 version 1.1.26 (Bates et al., 2015). The *p*‐values were calculated by comparing the full model with a null model excluding fixed effects, using a log‐likelihood test (Barr et al., 2013), and variance explained was estimated as marginal and conditional *r* ^2^ values (Nakagawa & Schielzeth, 2013) using mumin version 1.43.17 (Bartoń, 2020). *Post hoc* tests comparing factor levels used the Kenward–Rogers method in lsmeans version 2.30.0 and pbkrtest version 0.5.0.1 (Halekoh & Højsgaard, 2014; Lenth, 2016).

Estimating species richness notoriously undercounts the true richness in ecological studies, which is only compounded when rarifying data by the smallest sample size in a given study (Colwell et al., 2012). Methods to alleviate these problems have faced novel challenges with microbial data sets using high‐throughput sequencing due to sequencing errors being indistinguishable from novel taxa (Chiu & Chao, 2016). To alleviate these problems, species accumulation curves and estimated species richness were calculated with inext.3d version 1.0.1, which extrapolates species richness based on individual sample sizes using unrarefied data (Chao et al., 2014, 2021; Hsieh et al., 2016). Confidence intervals in inext.3d were calculated from 1,000 bootstrap replications. To assess functional potential, OTUs were assigned to functional guilds using funguild version 1.1 (N. H. Nguyen et al., 2016). Guild assignments were only kept if they reached the “Probable” and “Highly Probable” confidence levels. As each OTU could be assigned to multiple functional guilds, all functional guild assignments were counted to determine the proportional functional potential for each sample (i.e., if an OTU was assigned “Plant Pathogen” and “Saprotroph” it would be counted in both categories). A linear mixed effects model was used to test for temporal shifts in the proportional abundances of taxa in settled dust samples assigned to the “Plant Pathogen” functional guild, with month as a fixed effect and site as a random effect. The *p*‐values and *r* ^2^ values were calculated as described above. Visualizations were created using ggplot2 version 3.3.2 (Wickham, 2016). No novel code was used to perform the statistical analyses done in this study, though the code used has been included as Appendix S1.

## 3 RESULTS

A total of 1,002 individual soil and air samples were collected and their mycobiota sequenced and characterized using the ITS2 region of fungal ribosomal DNA, with 413 samples from Hwy33 sites and 589 samples from KARE, inclusive of previously published data (Gao et al., 2018, 2020). Hwy33 sites included 175 air and 238 soil samples, while KARE sites included 90 air and 499 soil samples. In total, 1,417 known fungal species (noninclusive of unidentified taxa at higher taxonomic levels) were identified from roughly 44,000,000 reads. The highest number of species was found in Hwy33 (930) and KARE (660) air samples, followed by Hwy33 (563) and KARE (499) soil samples (Figure S2, Table S3). The number of species found along Hwy33 did not differ significantly between individual sites, though there were more species found in air than in soil (Figure S3, Table S4). Of the total number of identified fungal species, slightly less than half (626 species) were unique to individual land use and sampling medium combinations. The number of sequence reads assigned to these species was quite small, however, representing only 1.4% of the total number of reads across both land uses and in air and soil. The highest number of uniquely sampled species (270) were found in Hwy33 air and the fewest (95) in KARE air (Figure S4), representing 0.05% and 0.02% of total sequence reads, respectively. In total, 172 species were found in common across all sampling mediums and sites, that is, in both soil and air and at both Hwy33 and KARE, representing 87.9% of total sequence reads. Between Hwy33 and KARE, more species were shared in air (503) than in soil (265), though the proportion of sequences in each category was nearly identical at 92.4% and 92.1%, respectively.

### 3.1 Fungal community structure

The most interesting and unexpected result of our analyses is the similarity in fungal assemblages in air over distances as great as 160 km, compared to the distinct nature of soil fungal communities over the same distances. Using PCoA, all samples separated into three distinct categories representing Hwy33 soil, KARE soil, and a third category containing all air samples from both Hwy33 and KARE (Figure 1b). The difference in PCoA‐derived mean Bray–Curtis distance between soil and air fungal communities at any individual site was greater than the difference in air between any pair of sites (Figure 1c). In terms of individual predictors of fungal community structure, PERMANOVA showed that land use (Hwy33 vs. KARE) and sampling medium (soil vs. air) explained 18% and 10%, respectively, of the variance in fungal community structure, while month, year and differences between Hwy33 sites were weak predictors (Table 1). Taken together, interactions between factors represented 18% of the explained variance, but only when inclusive of month or sampling medium. All factors showed significant differences (*p* ≤ .001) between factor levels, probably because of the high number of samples (van der Laan et al., 2010), with variance explained delineating important predictors from inconsequential ones. In general, the fungal assemblage in air more closely resembled the fungal community in soils from Hwy33 than from KARE, based on *post hoc* PERMANOVAs (Table S5). Reducing species to only those shared between soil and settled dust did not substantially change the results of PERMANOVA or PCoA (Figure S5), though subsampling to account for an unbalanced sampling design or heterogeneous dispersion between factor levels greatly reduced the effect of land use from 18% to 10% of the variance explained (Figure S6). In both cases, land use and sampling medium remained the most important explanatory variables. Likewise, re‐analysis in the absence of soils from Hwy33 (Figure S7) or KARE (Figure S8), to test if results were influenced by differences in soil sampling methods, did not change our core findings. Rarifying data did not change our findings and sequencing depth differences between samples could only explain about 1% of the total variance observed (Tables S6 and S7).

**TABLE 1:** PERMANOVA coefficient table for the Bray–Curtis dissimilarity among samples as a function of land use, site, year, month and sampling medium and the interactions between them in a fully nested model (adonis2 function).

Model (adonis2 function): ~ Land Use + Site + Year + Month + Medium + Medium*Land Use/Site/Year/Month					
	*df*	Sum of squares	*r* ^2^	*F*	*p* value
Land Use	1	64.74	.18	381.06	.001
Site	4	7.73	.02	11.38	.001
Year	1	12.08	.03	71.11	.001
Month	11	17.6	.05	9.42	.001
Medium	1	35.95	.10	211.58	.001
Land Use: Medium	1	15.56	.04	91.58	.001
Land Use: Site: Medium	4	4.62	.01	6.79	.001
Land Use: Site: Year: Medium	10	5.15	.01	3.03	.001
Land Use: Site: Year: Month: Medium	104	41.56	.12	2.35	.001
Residual	864	146.79	.42		
Total	1,001	351.78	1.00		

*Note*: Permutations = 1,000 (unstratified). *n* = 1002. *df* = degrees of freedom. *F* = pseudo F‐ratio (Anderson, 2001). Note: very low *p*‐values are probably a result of greatly increased sensitivity due to high replication (van der Laan et al., 2010), whereas *r* ^2^ and *F* values can better differentiate between important and trivial independent variables.

### 3.2 Spatial and temporal distance–decay

**FIGURE 2:**
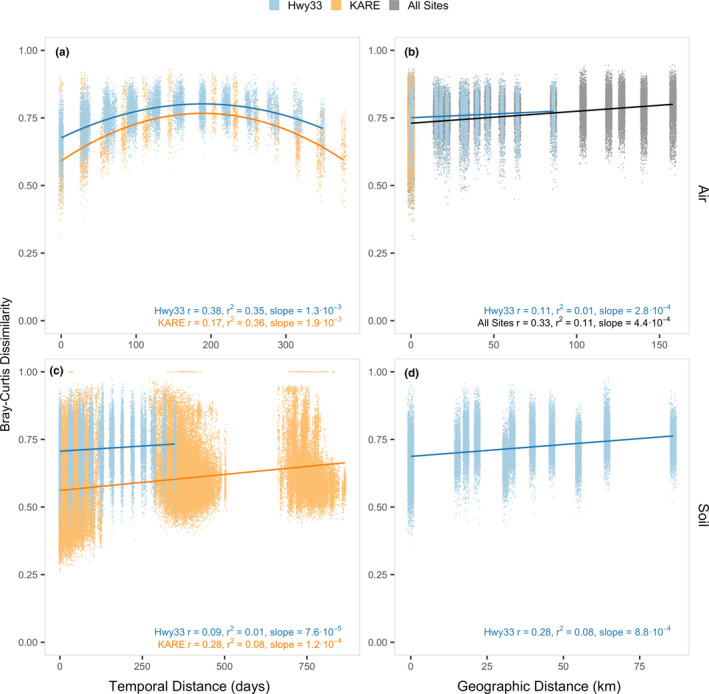
Effect of geographical distance and temporal distance on the composition of fungal communities. Relationships are between Bray–Curtis dissimilarity in air (a, b) and soil (c, d) samples. Temporal distance showed a stronger annual pattern in air (a) than in soils (c), while geographical distance (and land‐use change) showed little difference across air samples (b). Likewise, geographical distance among Hwy33 air samples was small compared to moderate differences between soil samples (d). *r* = Mantel statistic; *r* ^2^ = linear model coefficient of determination. The linear models for Hwy33 air and KARE air (a) both use a second‐order polynomial, and the reported slope is the initial rate of change. Geographical distance decay between soils (d) excludes KARE because of differences in sampling methods between land use types. Mantel *p* < .001 in all cases. Points are jittered up to ± 3 units on the *x*‐axis for visibility. Note: *x*‐axis range differs between panels.

Significant patterns of temporal and geographical distance–decay, with Bray–Curtis dissimilarity, were found for fungi in both Hwy33 and KARE samples, and in both the air and the soil (in all cases, Mantel *p* = .001) (Figure 2). In air samples, a seasonal pattern of fungal community dissimilarity and temporal distance was evident from the similar parabolic succession relationship seen at both Hwy33 (*r* ^2^ = .35, Mantel *r* = .38) and KARE (*r* ^2^ = .36, Mantel *r* = .17), with the initial rate of change significantly greater at KARE than at Hwy33 (*p* < .001) (Figure 2a). In contrast, the relationship between fungal community dissimilarity and geographical distance in air was very weak across Hwy33 sites (*r* ^2^ = .01, Mantel *r* = .11) over a maximum distance of ~80 km. When airborne fungi from KARE were included in the relationship, which were collected 100–160 km from Hwy33 sites, the slope of the relationship between community dissimilarity and geographical distance in air samples significantly increased (*p* < .001) by 36.4% when compared to Hwy33 sites alone (*r* ^2^ = .11, Mantel *r* = .33) (Figure 2b). Within Hwy33 sites, air samples taken no more than 50 m apart showed no relationship between dissimilarity and geographical distance (*p* > .09, |Mantel r| < .09, *r* ^2^ < .01). The relationship between fungal community dissimilarity and temporal distance was weaker in soils than in air at KARE (*r* ^2^ = .08, Mantel *r* = .28), and much weaker at Hwy33 sites (*r* ^2^ = .01, Mantel *r* = .09) (Figure 2c). The difference in temporal decay in soils between KARE and Hwy33 sites is almost certainly due to the former being actively cultivated agricultural land with regular seasonal disturbances due to planting, fertilization, irrigation and harvesting of crops. The temporal distance–decay relationship in soils was also analysed separately for each year at KARE, which all had significantly different slopes from one another (*p* < .001) (Figure S9). Bray–Curtis dissimilarity correlated significantly with temporal distance in KARE soils in 2016 and 2017 (Mantel *p* = .001) but not in 2018 (Mantel *p* = .7). This difference may be due to substantially fewer samples being sequenced and a shorter length of time investigated in 2018 (*n* = = 98, 4 months) than from 2016 (*n* = 254, 5 months) and 2017 (*n* = 147, 5 months). With geographical distance, the slope of the relationship with fungal community dissimilarity in soils over ~80 km along Hwy33 sites (*r* ^2^ = .08, Mantel *r* = .28) was significantly (*p* < .001) greater (~2.5‐fold) than in air (*r* ^2^ = .01, Mantel *r* = .11) (Figure 2d).

### 3.3 Functional guilds and taxonomy

**FIGURE 3:**
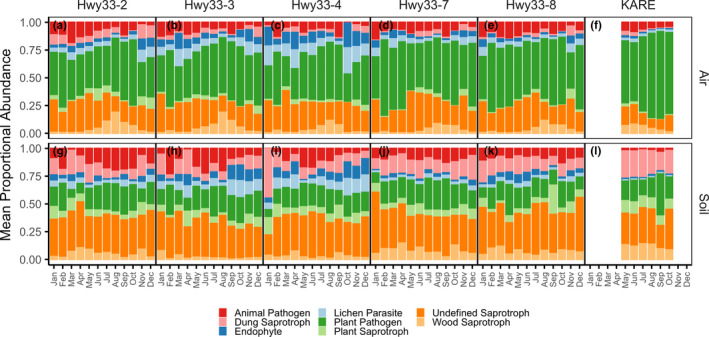
Mean proportional abundance of fungal guilds as a function of month, site and sampling medium. All guilds assigned to multiguild taxa were counted. Only guilds representing at least 1% of the community across all samples were included. Only funguild version 1.1 “Probable” and “Highly Probable” guild assignments were used. Note that November and December (2017) precede January–October (2018) for Hwy33.

**FIGURE 4:**
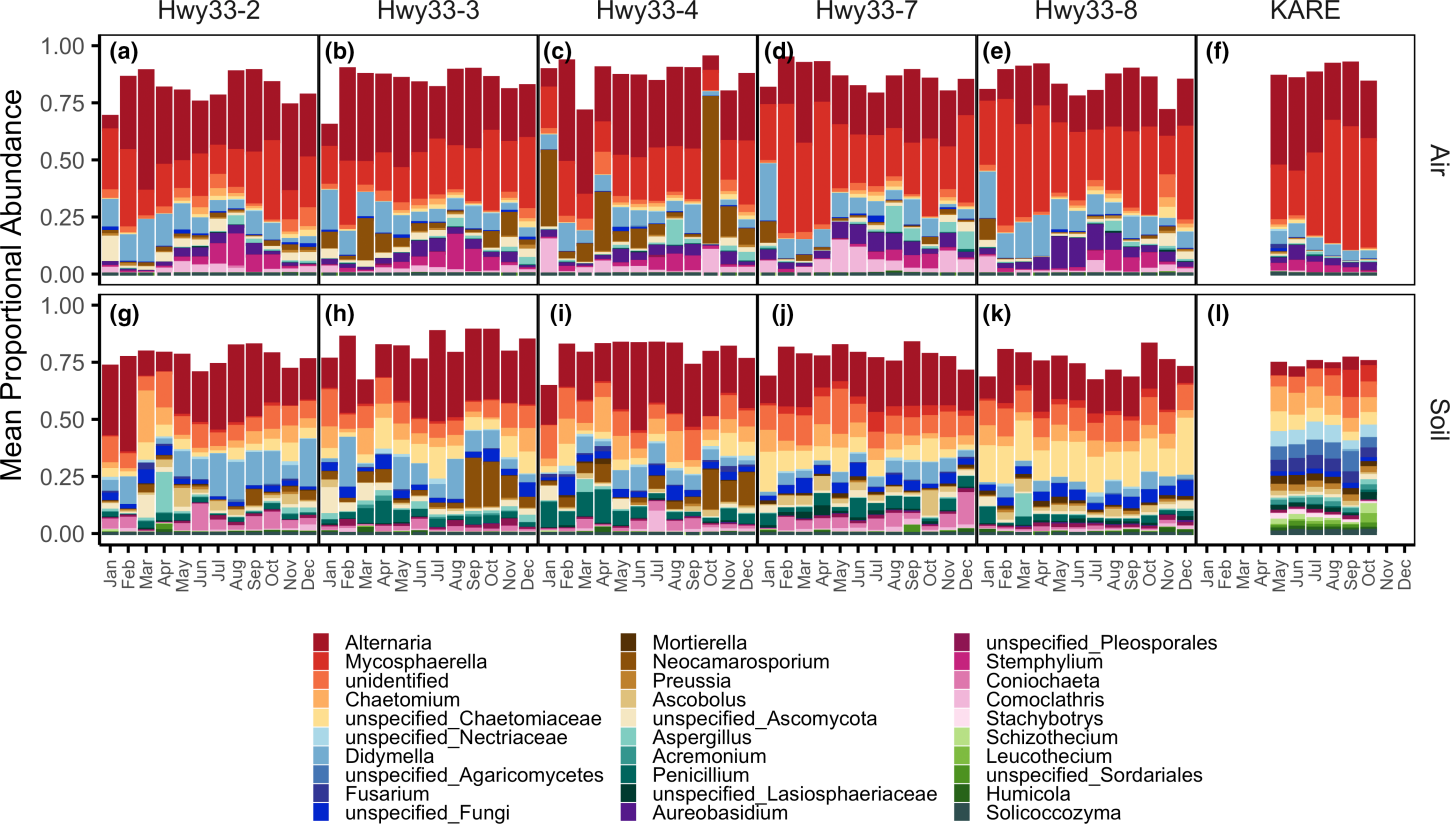
Mean proportional abundance of the top 30 most abundant genera, among all genera, as a function of month, site and sampling medium. Values are means between replicates, and across years (for KARE samples). unidentified = all pooled genera matching an unidentified reference sequence; unspecified = sequences binned into a taxonomic level without a reference sequence. Note that November and December (2017) precede January–October (2018) for Hwy33.

The distribution of functional guild assignments showed distinct sampling medium‐ and land‐use‐specific patterns (Figure 3). Air samples were dominated by the plant pathogen functional guild both along Hwy33 (46.7 ± 0.2%) and at KARE (71.2 ± 1.2%), while soil samples were dominated by the saprotroph functional guild, also both along Hwy33 (59.2 ± 0.9%) and at KARE (73.9 ± 0.8%) The proportional abundance of taxa assigned to the plant pathogen functional guild in air increased significantly from May to October (*p* < .001), though this pattern was less clear during the rest of the year (Figure S10). Plant pathogen functional guild percentages in soils were much lower than in air samples and were similar between soils at Hwy33 (20.1 ± 0.5%) and KARE (22.3 ± 0.8%). The animal pathogen functional guild, alternatively, was most abundant in Hwy33 soils (10.3 ± 0.5%), followed by Hwy33 air (8.6 ± 0.3%) and KARE air (6.6 ± 0.3%), and lowest in KARE soils (2.3 ± 0.3%). Taxonomic proportional abundances were characterized at the phylum, order and genus levels. Most taxa were Ascomycota in the Pleosporales, Capnodiales and Sordariales (Figures S11 and S12). The most common genera in air were *Mycosphaerella* (30.8%) and *Alternaria* (27.9%), which contain numerous plant and crop pathogenic species (Figure 4a–f). *Alternaria* was also the most common genus in soil fungi, but was much more common along Hwy33 (22.2%) than at KARE (9.6%) (Figure 4g–l). Genera of Onygenales, the order that contains *Coccidioides* as well as numerous animal pathogenic fungi (Sigler, 2002), were orders of magnitude more abundant in soils than in the air at both Hwy33 and KARE but did not differ significantly in proportional abundance between soils from Hwy33 and KARE, or between air samples from Hwy33 and KARE (Figure S13). *Coccidioides* was identified in only four soil samples from Hwy33 rodent burrows and was not found in any soil cores collected at KARE nor in any air samples. The soil samples that *Coccidioides* was detected in did not have an unusually high or low sequencing depth (Figure S14A), nor were the number of reads assigned to *Coccidioides* exceptional when compared to other taxa (Figure S14B).

## 4 DISCUSSION

In the study presented here, we investigated the assemblage of airborne fungi in settled dust and compared it to the soil fungal community in the most productive agricultural region in the United States, the SJV in California. Our study is one of only a handful to simultaneously compare the mycobiome in soil and air using high‐throughput sequencing. We showed that the assemblage of airborne fungi collected on both agricultural and undeveloped land, at distances of up to 160 km, resemble one another far more than they resemble the fungal communities in nearby soils. We also showed that, regardless of sampling location, the airborne fungal assemblage in the SJV was more similar to the fungal community in rodent burrow soils on undeveloped land than to the fungal community in agricultural soils. The similarity of the airborne fungal community across the SJV, though previously undocumented, is not entirely unexpected. Once airborne, fungal spores can be dispersed across vast distances (Barberán et al., 2015; Cáliz et al., 2018; Griffin, 2007), and the distribution of airborne fungal taxa can change little over tens (Kivlin et al., 2014) to hundreds (Nicolaisen et al., 2017) of kilometres and along altitudinal gradients of up to 1,000 m (Sánchez‐Parra et al., 2021). This degree of mixing is not always the case, however. Airborne fungi in Finnish conifer forests differ from one another at sites ~100–400 km distant from one another (Abrego et al., 2018), and to a lesser degree at distances as short as 1 km when sampling across a land‐use gradient between forested and urban areas (Abrego et al., 2020). In the coniferous forest study, airborne fungal assemblages from hundreds of kilometres away were more similar to one another than to soil fungi previously characterized at the same sites (Abrego et al., 2018; Mäkipää et al., 2017). These results raise the possibility that the findings we present may not be isolated only to arid environments such as the SJV, but are instead relevant across multiple biomes.

### 4.1 Spatial and temporal patterns

Distance–decay relationships are helpful for understanding patterns in community ecology (Anderson et al., 2011; Dray et al., 2012; Nekola & White, 1999; Soininen et al., 2007; Whittaker, 1972). Among studies of fungi, such relationships can illustrate both geographical variation (Bahram et al., 2013; Barberán et al., 2015) and patterns of dispersal limitation (Adams et al., 2013; Peay et al., 2012), both of which are relevant to the current study. The fungi that we sampled in soil and in settled dust showed evidence of dispersal limitation based on significant correlations between Bray–Curtis dissimilarity of the fungal community and geographical distance. Dispersal limitation of airborne fungi has been reported previously in settled dust (Adams et al., 2013; Barberán et al., 2015) and rain spore traps (Peay et al., 2012). Conversely, airborne fungi sampled in southern California showed no evidence of dispersal limitation (Kivlin et al., 2014). The reasons for this difference in findings are probably methodological: Kivlin et al. (2014) sequenced the 18S region of fungal rDNA, which provides a decidedly lower species‐level resolution than the ITS2 region (Bruns & Taylor, 2016; Schoch et al., 2012) and used an older sequencing technology than the one we used. However, differences may also be related to the frequency and scale of sampling, the geographical region investigated or the sampling methods employed, all of which can influence distance–decay relationships (Clark et al., 2021; Soininen et al., 2007). Our finding that the distance–decay relationship was significantly stronger for soil fungi than airborne fungi reflects our observations of greater community variation in soil fungi than in airborne fungi in the SJV. This result provides support for our main finding that the air mycobiome is more similar than the soil mycobiome, not only with regard to ecological distance, but when incorporating a physical measure of distance as well. While we could assess the distance–decay relationship in soils over the 80 km separating undeveloped sites along Hwy33, we felt this relationship could not be extended to soils at KARE due to differences in land management, sampling methods and an absence of rodent burrows on agricultural land. However, the environmental conditions at our undeveloped sites are largely representative of a substantial portion of the undeveloped land in the SJV (Griffith et al., 2016), indicating that our findings regarding the soil fungal community may be generalizable at a larger landscape scale. This point is supported by the fact that rodent burrows, such as the ones we sampled, are exceedingly common across similar arid environments (Davidson & Lightfoot, 2008; Grinnell, 1923; Whitford & Kay, 1999).

As previously noted, the airborne fungi surveyed here and in other studies that use molecular identification techniques are not required to establish and grow (Adams et al., 2013). What is measured is only the DNA that is associated, or was associated, with a living organism. Environmental stressors in the atmosphere, such as ultraviolet irradiation and desiccation, can render airborne fungal spores nonviable (Griffin, 2004; Ulevičius et al., 2004), though dormancy mechanisms can confer a fitness advantage by protecting against such stressors (Nemergut et al., 2013). However, this selective force remains unmeasured, probably inflating the perceived diversity of viable airborne fungi across geographical distances, and possibly underestimating airborne fungal dispersal limitation. The influence of nonviable fungi that plagues studies of airborne fungi is lessened in soils, where unprotected nucleic acids are subject to decomposition (Gordon & Van Norman, 2021). Still, some fungal spores can persist in soils for many years, confusing the detection of growing fungi with dormant fungi (Aime & Miller Jr, 2002; Bruns et al., 2009; Nguyen, 2018; Sussman et al., 1966). The germination and growth of fungal spores in soils, unlike in air and settled dust, raises the prospect that fitness advantages conferred by dormancy mechanisms and favourable adaptations to local edaphic conditions contribute to the observed community structure.

The seasonal pattern (temporal‐decay) we observed in airborne fungi in the SJV has been reported from other studies based on abundances of individual fungal taxa in air (Almaguer‐Chávez et al., 2012; Lacey, 1981; Lagomarsino Oneto et al., 2020; Reyes et al., 2016). Likewise, the distribution of taxa that make up the outdoor airborne fungal assemblage is associated with the frequency and timing of sample collection, whether weekly, seasonally or yearly (Cáliz et al., 2018; Du et al., 2018; Fierer et al., 2008; Nicolaisen et al., 2017). We hypothesize that the seasonal pattern we observed in airborne fungi is due to the annual, agricultural cycle of planting and harvesting, whereupon crops are generally planted in the spring and harvested in the autumn (Zhong et al., 2011), as well as the yearly phenology of wild plants in the SJV (Chiariello, 1989). This hypothesis is supported by our observations of monthly shifts in the proportional abundance of taxa assigned to the plant pathogen functional guild as well as the dominance of *Alternaria* and *Mycosphaerella* in settled dust samples, genera that contain numerous plant pathogenic and parasitic species (Camiletti et al., 2022; Crous, 2010; Farrar et al., 2004; Fones et al., 2020; Koike et al., 2017). Similar seasonal patterns to the ones we show here have been observed in other agricultural regions, with increased abundances of plant pathogenic fungi in the late summer and autumn (Almaguer‐Chávez et al., 2012; Nicolaisen et al., 2017). The soil fungal community in the SJV, in contrast to the airborne fungal assemblage, changed little with time, suggesting that shifts in the distribution of fungi present in soils cannot fully explain the corresponding shifts in the distribution of airborne fungi above. While fungi inhabiting soil can survive adverse conditions as vegetative hyphae, through the production of sclerotia (Willetts, 1971) or as spore banks (Baar et al., 1999), fungi inhabiting living tissue on the aerial structures of host plants typically must sporulate for persistence. The high abundance of *Mycosphaerella* in air samples, and its relative near absence in soil samples, indicates that this genus is largely unassociated with soils in the SJV. We presume that airborne *Mycosphaerella*, which constitutes over half of the air mycobiome in some land‐use and month combinations, as well as other plant pathogenic and parasitic fungi, are more associated with the crop and wild plant phyllosphere than the soil environment in the SJV.

### 4.2 Land use and the influence of burrowing rodents

Our results indicate that most airborne fungal taxa in the SJV can be found in both agricultural soils and soils within rodent burrows on undeveloped land. Mean Bray–Curtis dissimilarities and proportional abundances of taxa indicate that soils from rodent burrows more closely resemble the air mycobiome in the SJV than soils from agricultural fields. Though we believe that the air mycobiome in the SJV is probably more associated with plants than with soils, at some point in their life cycle most described fungi can be found in soils (Bridge & Spooner, 2001; O'Brien et al., 2005; Tedersoo et al., 2014). Fungi primarily disperse through the air (Magyar et al., 2016; Talbot, 1997), and in arid environments, wind erosion can liberate large volumes of surface soil and dust (Duniway et al., 2019; Field et al., 2010), probably dispersing fungi and fungal spores (Barberán et al., 2015; Dietzel et al., 2019; Schiro et al., 2022). This type of dispersal suggests that, though perhaps not the dominant source of airborne fungi, soil fungi and their spores can contribute significantly to the air mycobiome in arid environments such as those found in the SJV. While the SJV is generally considered an arid environment (Griffith et al., 2016), the physical characteristics of cultivated agricultural land within the SJV are probably less susceptible to wind disturbance than undeveloped land due to artificial irrigation and crop cover (Duniway et al., 2019). Conversely, on undeveloped lands, burrowing mammals can liberate significant quantities of fine soil material (Black & Montgomery, 1991; Davidson & Lightfoot, 2008; Grinnell, 1923; Whitford & Kay, 1999), and this material is highly susceptible to wind erosion (Wei et al., 2007; Whitford & Kay, 1999). It has been estimated that, in areas where foraging occurs, up to 20% of the soil surface is disturbed by burrowing mammals in arid environments each year (Whitford & Kay, 1999). The data we have collected do not allow us to determine the origin of the airborne fungi we found in the SJV, as many variables were left unexplored and the range of locations sampled was limited. However, the susceptibility of soils from rodent burrows to wind erosion could offer a plausible starting point for explaining the higher similarity we found between airborne fungi and fungi from rodent burrow soils, than fungi from agricultural soils.

### 4.3 Methodological considerations

An important question is to what degree sampling method and study site selection play a role in the observed distribution of fungal species. There are numerous methods for sampling airborne fungi, each with its own unique trade‐offs (West & Kimber, 2015). We used passive deposition sampling on Petri dishes, which is inexpensive and allows for a high degree of replication. Differences in spore aerodynamic diameter, however, may enrich for specific taxa with larger spores and higher settling velocities when using deposition sampling (C. Woo et al., 2018). Kivlin et al. (2014) sampled airborne fungi on nylon filters that use an active air pump, which can probably capture a wider distribution of particle sizes. However, filters on active samplers such as this slowly become clogged with material (West & Kimber, 2015), which may impact the distribution of fungi sampled over time. Indeed, Kivlin et al. (2014) state that filter replacement was sometimes necessitated due to obstructed airflow. A bigger drawback is the cost of active sampling systems and their need for electricity, which limits replication. For example, our passive sampling method allowed for monthly collection from 13 samplers at KARE for three summers, and 15 samplers among the five Hwy33 sites for one year, whereas Kivlin et al. (2014) collected one filter from each of five sites every 2–3 months, over a 17‐month period. In both studies by Abrego et al. (2018, 2020), a “cyclone sampler” was used, which probably captures the most representative sample of airborne fungi of the methods mentioned (Abrego et al., 2018), though replication is limited by cost similar to filtration methods. An important consideration regarding the results of ours and the few other metabarcoding studies that have compared airborne fungi and soil fungi is that sampling methods between these two mediums are not equivalent. While the soil fungal community may have taken years to arrive at its current state (Osburn et al., 2021), the airborne fungal assemblage can change with the seasons, as we have shown here. There are probably numerous other differences between these sampling mediums with which to contend, and while we are currently unable to address all of them, it is important to evaluate our results with this caveat in mind.

The location in the environment where air sampling takes place, particularly sampling height, can influence the distribution of fungal taxa observed (Charalampopoulos et al., 2022; Khattab & Levetin, 2008; Mahaffee, 2014). Sampling closer to the ground (0.5–1.5 m) better represents local taxonomic distributions of airborne fungi while sampling higher up (10–30 m) is more likely to represent regional distributions (Lacey & Venette, 1995; West & Kimber, 2015). Sampling very high up (>100 m) appears to homogenize fungal aerobiota (Núñez & Moreno, 2020; Sánchez‐Parra et al., 2021; Tipton et al., 2019). Both our study and those of Abrego et al. (2018, 2020) sampled close to the soil surface and found small but significant differences in the distribution of airborne taxa between sites. It is possible that our study and Abrego et al. (2018, 2020) preferentially sampled more localized fungal taxa. Sampling at low heights near the saltation layer (the height range where wind causes particles to skip across the soil surface) enriches for particles from the surrounding area (Ho et al., 2014; Martin & Kok, 2017). In contrast, Kivlin et al. (2014) sampled at 7 m and found no significant differences among sites or between seasons, while we showed clear differences with both. Though Kivlin et al. (2014) used different sequencing methods than ours, it is possible that sampling at a greater height also homogenized the distribution of fungi sampled with respect to time as well as location. This possibility is supported by fungal sampling at elevations above 3, 000 m in which the distribution of airborne fungal taxa can become completely decoupled with time from the seasonal to decadal scale (Tipton et al., 2019).

### 4.4 Difficulty in detecting *Coccidioides*

Our inability to detect *Coccidioides* in all but four samples prevents us from saying much that is ecologically relevant regarding *Coccidioides* in either soil or air. It is notable however that the sequencing depth and the total number of reads assigned to *Coccidioides* in samples where *Coccidioides* was detected were neither remarkably high nor low. This suggests that the likelihood of finding *Coccidioides* in soils may be more associated with an uneven distribution across the landscape (Greene et al., 2000; Maddy, 1958; Stewart & Meyer, 1932) rather than its presence at some minimum abundance. Detecting *Coccidioides* in air samples has only been accomplished three times from ambient air (Ajello et al., 1965; Daniels et al., 2002; Gade et al., 2020), and once from dust generated through disturbance of the soil surface with a leaf blower (Chow et al., 2016). In all three cases, a high‐throughput pump was used to sample thousands of litres of air, which contrasts greatly with our passive deposition sampling method. It is possible that our sampling method did not allow for the reliable detection of *Coccidioides*, which may only be present in the air in extremely small abundances. Our finding that fungi in the order Onygenales are far more common in soils than in settled dust, on both agricultural and undeveloped land, allows for speculation that soil disturbance may be important for dispersal and infection of animal pathogenic fungi (including *Coccidioides*). The higher proportional abundance of taxa assigned to the animal pathogenic fungal guild in the air and soil of undisturbed land than those of agricultural land hints at possible source dynamics, though more work is needed here. Regardless, high‐throughput air sampling techniques should be used in any future attempts to capture airborne *Coccidioides* fungi and a more sensitive *Coccidioides* detection strategy, such as using the CocciENV qPCR assay (Bowers et al., 2019) should be applied in future work investigating *Coccidioides* in either soil or air.

## 5 CONCLUSION

The study presented here provides an analysis of the most extensive sampling effort of fungi in both soil and air to date using high‐throughput sequencing methods. By comparing the settled dust mycobiome with the spatially associated soil mycobiome from two distinct sources (rodent burrows on undeveloped land and soil cores from agricultural land), we show that the airborne fungal assemblage in the SJV in California is far more similar between sites over 100 km away than to nearby soil fungal communities. Our results indicate that the air mycobiome in the SJV experiences seasonal cycles which we hypothesize are the result of the cultivation of crop plants on agricultural land and the phenology of wild plants. We show that, despite the relative similarity of the air mycobiome among sites when compared to the soil mycobiome, significant geographical patterns are apparent. This pattern is elucidated most clearly through the evidence we provide for airborne fungal dispersal limitation in the SJV. Finally, we hypothesize that the broad array of methodological differences used to explore airborne fungi in the past are probably responsible for differences in results, and that future work should seek to either standardize methods or present results in the context of the methods used. Taken together, our study provides an important exploration of airborne fungal dispersal in the SJV in California, which will be important for gaining a better understand of how fungal pathogens spread in the outdoor environment. This information will be useful in helping to prevent airborne fungal disease as well as in providing a broader understanding of fungal community ecology.

## CONFLICTS OF INTEREST

The authors have no conflicts of interest.

### OPEN RESEARCH BADGES

This article has earned an Open Data badge for making publicly available the digitally‐shareable data necessary to reproduce the reported results. The data is deposited in the NCBI Sequence Read Archive (www.ncbi.nlm.nih.gov/sra) with the following accession numbers: PRJNA736519, PRJNA736167 and PRJNA736543.

## DATA ACCESSIBILITY STATEMENT

All data supporting the findings of this study have been deposited in the NCBI Sequence Read Archive (www.ncbi.nlm.nih.gov/sra) with the following accession numbers: PRJNA736519, PRJNA736167 and PRJNA736543. All metadata, bioinformatics code and statistical code needed to replicate this study are included as Appendix S1.

## AUTHOR CONTRIBUTIONS

Robert Wagner and John W. Taylor designed the research approach while Justin Remais, Jennifer R. Head and John W. Taylor initially conceived of the project. Robert Wagner, John W. Taylor, Cheng Gao and Liliam Montoya collected and analyzed samples and Robert Wagner performed all statistical analyses, generated all visualizations and directed the structure of the text. All authors contributed to the writing and editing of the final manuscript.

## BENEFIT‐SHARING

The people living in the region from which samples were collected will benefit through a better understanding of the ecological dynamics of local airborne fungal pathogens. This understanding can inform prevention and mitigation strategies regarding fungal pathogens of humans, such as *Coccidioides*, as well as fungal pathogens of crops, wild plants, and domesticated and wild animals in the region studied.

## Supporting information


Appendix S1

Appendix S2
