# Supplementary material for: The air mycobiome is decoupled from the soil mycobiome in the California San Joaquin Valley
Source: Mol Ecol. 2022 Aug 25;31(19):4962–78. doi: 10.1111/mec.16640 (PMC9624177; doi:10.1111/mec.16640)
Supplement: Supplementary file 1 — Appendix S1 [file MEC-31-4962-s002.zip › MEC_16640_2022.07.21.bioinformatics.code.pdf]

MOLECULAR ECOLOGY

Bioinformatics Code for:

The air mycobiome is decoupled from the soil mycobiome in the California San Joaquin Valley

Robert Wagner, Liliam Montoya, Cheng Gao, Jennifer R. Head, Justin Remais, John W. Taylor

```
# import FASTAs into artifact object (make sure to set the correct path!)
qiime tools import --type 'SampleData[PairedEndSequencesWithQuality]' --
input-path /fastq --input-format CasavaOneEightSingleLanePerSampleDirFmt -
-output-path demux.qza

# generate summary
qiime demux summarize --i-data demux.qza --o-visualization demux.qzv
qiime tools view demux.qzv

# denoise with dada2 (truncate read ends)
# Forward primer = 5.8s-Fun (AACTTT...CAA.GGATC.CT)
# Reverse primer = ITS4-Fun (AGCCTCCGCTTATTGATATGCTTAA.T)
# Quality cutoff at >= 25
# (trim parameters below differ between runs but adhere to the quality
# cutoff)
qiime dada2 denoise-paired --i-demultiplexed-seqs demux.qza --p-trim-left-
f 22 --p-trunc-len-f 300 --p-trim-left-r 28 --p-trunc-len-r 258 --p-n-
threads 7 --o-representative-sequences rep-seqs-dada2.qza --o-table table-
dada2.qza --o-denoising-stats stats-dada2.qza

# generate visualization for denoise
qiime metadata tabulate --m-input-file stats-dada2.qza --o-visualization
stats-dada2.qzv
qiime tools view stats-dada2.qzv

# rename files
mv rep-seqs-dada2.qza rep-seqs.qza
mv table-dada2.qza table.qza

# import UNITE database
qiime tools import --type 'FeatureData[Sequence]' --input-path
sh_refs_qiime_ver8_97_s_02.02.2019.fasta --output-path reference.qza
qiime tools import --type 'FeatureData[Taxonomy]' --input-format
HeaderlessTSVTaxonomyFormat --input-path
sh_taxonomy_qiime_ver8_97_s_02.02.2019.txt --output-path taxonomy.qza

# train naive bayes classifier
qiime feature-classifier fit-classifier-naive-bayes --i-reference-reads
reference.qza --i-reference-taxonomy taxonomy.qza --o-classifier
classifier.qza

# run sklearn naive bayes classifier
qiime feature-classifier classify-sklearn --verbose --p-n-jobs 7 --i-
classifier classifier.qza --i-reads rep-seqs.qza --o-classification
taxonomy-output.qza

qiime metadata tabulate \
  --m-input-file taxonomy-output.qza \
  --o-visualization taxonomy-output.qzv

qiime tools view taxonomy-output.qzv

# collapse taxa table
```

```
qiime taxa collapse --i-table table.qza --i-taxonomy taxonomy-output.qza -
-p-level 7 --o-collapsed-table table-collapsed.qza

# export fearture table to biom file and convert biom file to tsv
qiime tools export \
  --input-path table-collapsed.qza \
  --output-path exported-feature-table
cd exported-feature-table
biom convert -i feature-table.biom -o feature-table.tsv --to-tsv




### R code ###

# next, open the tsv file in R and make taxa tables at each taxonomic
level.

# load libraries
library(reshape2)

# load otu data
d.otus = read.table("feature-table.tsv", sep = "\t", skip = 1,
comment.char = "")

d.otus = t(d.otus)
colnames(d.otus) = d.otus[1,]
colnames(d.otus)[1] = "sample"
d.otus = d.otus[-1,]
d.otus = as.data.frame(d.otus)
d.otus = sapply(d.otus, as.numeric)
d.otus = as.data.frame(d.otus)

# melt otu data into long format
d.melt = melt(d.otus, id=c("sample"))

# remove taxa level prefix from otu names
d.melt$variable = gsub("[a-z]__", "", d.melt$variable)

# make kingdom column
function.kingdom = function(x) substr(x, 1, unlist(gregexpr(";", x))[1] -
1)
d.melt$kingdom = lapply(d.melt$variable, function.kingdom)

# make phylum column
function.phylum = function(x) substr(x, unlist(gregexpr(";", x))[1] + 1,
unlist(gregexpr(";", x))[2] - 1)
d.melt$phylum = unlist(lapply(d.melt$variable, function.phylum))

# make class column
function.class = function(x) substr(x, unlist(gregexpr(";", x))[2] + 1,
unlist(gregexpr(";", x))[3] - 1)
d.melt$class = unlist(lapply(d.melt$variable, function.class))
```

```
# make order column
function.order = function(x) substr(x, unlist(gregexpr(";", x))[3] + 1,
unlist(gregexpr(";", x))[4] - 1)
d.melt$order = unlist(lapply(d.melt$variable, function.order))

# make family column
function.family = function(x) substr(x, unlist(gregexpr(";", x))[4] + 1,
unlist(gregexpr(";", x))[5] - 1)
d.melt$family = unlist(lapply(d.melt$variable, function.family))

# make genus column
function.genus = function(x) substr(x, unlist(gregexpr(";", x))[5] + 1,
unlist(gregexpr(";", x))[6] - 1)
d.melt$genus = unlist(lapply(d.melt$variable, function.genus))

# make species column
function.species= function(x) substr(x, unlist(gregexpr(";", x))[6] + 1,
nchar(x))
d.melt$species = unlist(lapply(d.melt$variable, function.species))

# replace __ wih Unknown
d.melt[d.melt == "__"] = "unspecified"

# append "unspecified" with next highest identified taxa level
unspecified.phylum = function (x) paste("unspecified_", substr(x, 1,
unlist(gregexpr(";", x))[1]-1), sep = "")
d.melt[d.melt$phylum=="unspecified",][,5:10] =
unlist(lapply(d.melt[d.melt$phylum=="unspecified",]$variable,
unspecified.phylum))

unspecified.class = function (x) paste("unspecified_", substr(x,
unlist(gregexpr(";", x))[1]+1, unlist(gregexpr(";", x))[2]-1), sep = "")
d.melt[d.melt$class=="unspecified",][,6:10] =
unlist(lapply(d.melt[d.melt$class=="unspecified",]$variable,
unspecified.class))

unspecified.order = function (x) paste("unspecified_", substr(x,
unlist(gregexpr(";", x))[2]+1, unlist(gregexpr(";", x))[3]-1), sep = "")
d.melt[d.melt$order=="unspecified",][,7:10] =
unlist(lapply(d.melt[d.melt$order=="unspecified",]$variable,
unspecified.order))

unspecified.family = function (x) paste("unspecified_", substr(x,
unlist(gregexpr(";", x))[3]+1, unlist(gregexpr(";", x))[4]-1), sep = "")
d.melt[d.melt$family=="unspecified",][,8:10] =
unlist(lapply(d.melt[d.melt$family=="unspecified",]$variable,
unspecified.family))

unspecified.genus = function (x) paste("unspecified_", substr(x,
unlist(gregexpr(";", x))[4]+1, unlist(gregexpr(";", x))[5]-1), sep = "")
d.melt[d.melt$genus=="unspecified",][,9:10] =
unlist(lapply(d.melt[d.melt$genus=="unspecified",]$variable,
unspecified.genus))
```

```
unspecified.species = function (x) paste("unspecified_", substr(x,
unlist(gregexpr(";", x))[5]+1, unlist(gregexpr(";", x))[6]-1), sep = "")
d.melt[d.melt$species=="unspecified",][,10] =
unlist(lapply(d.melt[d.melt$species=="unspecified",]$variable,
unspecified.species))

# cast molten data at each taxonomic level
d.cast.phylum = dcast(d.melt, sample ~ phylum, sum)
d.cast.class = dcast(d.melt, sample ~ class, sum)
d.cast.order = dcast(d.melt, sample ~ order, sum)
d.cast.family = dcast(d.melt, sample ~ family, sum)
d.cast.genus = dcast(d.melt, sample ~ genus, sum)
d.cast.species = dcast(d.melt, sample ~ species, sum)
```
