# Supplementary material for: The air mycobiome is decoupled from the soil mycobiome in the California San Joaquin Valley
Source: Mol Ecol. 2022 Aug 25;31(19):4962–78. doi: 10.1111/mec.16640 (PMC9624177; doi:10.1111/mec.16640)
Supplement: Supplementary file 1 — Appendix S1 [file MEC-31-4962-s002.zip › MEC_16640_2022.07.21.statistics.code.pdf]

MOLECULAR ECOLOGY

Statistics Code for:

The air mycobiome is decoupled from the soil mycobiome in the California San Joaquin Valley

Robert Wagner, Liliam Montoya, Cheng Gao, Jennifer R. Head, Justin Remais, John W. Taylor

```
### PCoA and PERMANOVA

# load libraries
library(vegan)
library(ape)
library(lsmeans)
library(psych)
library(data.table)

# load data
d = read.csv("d.combined.species.csv")

# remove unidentified and unspecified taxa
d = d[,-grep("unspecified", colnames(d))]
d = d[,-grep("unidentified", colnames(d))]

# remove rows containing no taxa
d = d[which(rowSums(d[,10:dim(d)[2]]) != 0),]

# load meta data
d.meta = d[,1:9]

# make data matrix
d.matrix = as.matrix(d[,10:dim(d)[2]])
# remove rare species (optional)
d.matrix.removed.rare = d.matrix[,colSums(d.matrix) > 1]
# first squareroot and wisconsin 2x transform data
d.matrix.transformed = sqrt(d.matrix.removed.rare)
d.matrix.transformed = wisconsin(d.matrix.transformed)
# next, create dissimilarity matrix with transformed data (bray-curtis)
d.dist = vegdist(d.matrix.transformed, method="bray")

# ordination (PCoA)
myPCoA = pcoa(d.dist)

# Permutational multivariate analysis of variance (PerMANOVA)
# note: Site = "Land Use", site = "Site", year = "Year", month = "Month",
type = "Medium"
adonis2(d.dist ~ Site + site + year + month + type +
Site/site/year/month/type, d.meta, parallel = 8, method = "bray",
permutations = 1000)

# Permutational multivariate analysis of variance (PerMANOVA) (with
strata[or "blocks"])
# https://github.com/vegandevs/vegan/issues/427
# https://stats.stackexchange.com/questions/350462/can-you-perform-a-
permanova-analysis-on-nested-data
# https://stats.stackexchange.com/questions/188519/adonis-in-vegan-order-
of-variables-or-use-of-strata/238962#238962

perm = how(nperm = 1000)
setBlocks(perm) = with(d.meta, type)
```

```
adonis2(d.dist ~ Site + site + year + month + type +
Site/site/year/month/type, d.meta, parallel = 8, method = "bray",
permutations = perm)



### Distance Decay

# load libraries
library(vegan)
library(ape)

# load data
d = read.csv("d.combined.species.csv")

# remove unidentified and unspecified taxa
d = d[,-grep("unspecified", colnames(d))]
d = d[,-grep("unidentified", colnames(d))]

# remove rows containing no taxa
d = d[which(rowSums(d[,10:dim(d)[2]]) != 0),]

# load meta data
d.meta = d[,1:9]

# make data matrix
d.matrix = as.matrix(d[,10:dim(d)[2]])
# remove rare species (optional)
d.matrix.removed.rare = d.matrix[,colSums(d.matrix) > 1]
# first squareroot and wisconsin 2x transform data
d.matrix.transformed = sqrt(d.matrix.removed.rare)
d.matrix.transformed = wisconsin(d.matrix.transformed)
# next, create dissimilarity matrix with transformed data (bray-curtis)
d.dist = vegdist(d.matrix.transformed, method="bray")

# create a dissimilarity matrix with transformed data
d.dist = vegdist(d.matrix.transformed, method="bray")
d.dist.air = vegdist(d.matrix.transformed[d.meta$type=="Air",],
method="bray")
d.dist.soil = vegdist(d.matrix.transformed[d.meta$type=="Soil",],
method="bray")
d.dist.hwy33.air = vegdist(d.matrix.transformed[d.meta$site!="KARE" &
d.meta$type=="Air",], method="bray")
d.dist.hwy33.soil = vegdist(d.matrix.transformed[d.meta$site!="KARE" &
d.meta$type=="Soil",], method="bray")
d.dist.kare.air = vegdist(d.matrix.transformed[d.meta$site=="KARE" &
d.meta$type=="Air",], method="bray")
d.dist.kare.soil = vegdist(d.matrix.transformed[d.meta$site=="KARE" &
d.meta$type=="Soil",], method="bray")

# make another site and type factor for all sites
d.meta$sitetype2 = as.factor(paste(d.meta$site, d.meta$type, sep = "-"))

# create latitude and longitude table by site
```

```
d.lat.long =
read.csv("/Users/user/Desktop/Cocci2020/data.meta/sites.lat.long.csv")
d.lat.long[d.lat.long$site!="KARE",]$site = paste("Hwy33-",
d.lat.long[d.lat.long$site!="KARE",]$site, sep = "")

d.geo = d.lat.long[match(d.meta$site, d.lat.long$site),]

# create euclidean distance matrix
d.dist.geo = vegdist(d.geo[,2:3], method="euclidean")*111
d.dist.geo.air = vegdist(d.geo[d.meta$type=="Air",2:3],
method="euclidean")*111
d.dist.geo.soil = vegdist(d.geo[d.meta$type=="Soil",2:3],
method="euclidean")*111
d.dist.geo.hwy33.air = vegdist(d.geo[d.meta$site!="KARE" &
d.meta$type=="Air",2:3], method="euclidean")*111
d.dist.geo.hwy33.soil = vegdist(d.geo[d.meta$site!="KARE" &
d.meta$type=="Soil",2:3], method="euclidean")*111
d.dist.geo.kare.air = vegdist(d.geo[d.meta$site=="KARE" &
d.meta$type=="Air",2:3], method="euclidean")*111
d.dist.geo.kare.soil = vegdist(d.geo[d.meta$site=="KARE" &
d.meta$type=="Soil",2:3], method="euclidean")*111

# create a vector of julian dates
d.meta$date = paste(d.meta$year, d.meta$month, d.meta$day, sep = "-")
date.samples = strptime(d.meta$date, "%Y-%B-%e")
d.time = as.integer(round(julian(date.samples), 0))

# create a temporal (euclidean) distance matrix from julian dates
d.dist.time = vegdist(d.time, method="euclidean")
d.dist.time.air = vegdist(d.time[d.meta$type=="Air"], method="euclidean")
d.dist.time.soil = vegdist(d.time[d.meta$type=="Soil"],
method="euclidean")
d.dist.time.hwy33.air = vegdist(d.time[d.meta$site!="KARE" &
d.meta$type=="Air"], method="euclidean")
d.dist.time.hwy33.soil = vegdist(d.time[d.meta$site!="KARE" &
d.meta$type=="Soil"], method="euclidean")
d.dist.time.kare.air = vegdist(d.time[d.meta$site=="KARE" &
d.meta$type=="Air"], method="euclidean")
d.dist.time.kare.soil = vegdist(d.time[d.meta$site=="KARE" &
d.meta$type=="Soil"], method="euclidean")



# air

# fit linear models for dist ~ time for air hwy33, air KARE and both
combined
fit.time.air.hwy33 = lm(d.dist.hwy33.air ~ d.dist.time.hwy33.air +
I(d.dist.time.hwy33.air^2))
fit.time.air.hwy33.linear = lm(d.dist.hwy33.air ~ d.dist.time.hwy33.air)
prd.time.air.hwy33 = data.frame(d.dist.time.hwy33.air = seq(from =
range(d.dist.time.hwy33.air)[1], to = range(d.dist.time.hwy33.air)[2],
length.out = 100))
```

```
err.time.air.hwy33 = predict(fit.time.air.hwy33, newdata =
prd.time.air.hwy33, se.fit = TRUE)

prd.time.air.hwy33$lci = err.time.air.hwy33$fit - 1.96 *
err.time.air.hwy33$se.fit
prd.time.air.hwy33$fit = err.time.air.hwy33$fit
prd.time.air.hwy33$uci = err.time.air.hwy33$fit + 1.96 *
err.time.air.hwy33$se.fit

fit.time.air.kare = lm(d.dist.kare.air ~ d.dist.time.kare.air +
I(d.dist.time.kare.air^2))
fit.time.air.kare.linear = lm(d.dist.kare.air ~ d.dist.time.kare.air)
prd.time.air.kare = data.frame(d.dist.time.kare.air = seq(from =
range(d.dist.time.kare.air)[1], to = range(d.dist.time.kare.air)[2],
length.out = 100))
err.time.air.kare = predict(fit.time.air.kare, newdata =
prd.time.air.kare, se.fit = TRUE)

prd.time.air.kare$lci = err.time.air.kare$fit - 1.96 *
err.time.air.kare$se.fit
prd.time.air.kare$fit = err.time.air.kare$fit
prd.time.air.kare$uci = err.time.air.kare$fit + 1.96 *
err.time.air.kare$se.fit

fit.time.air = lm(d.dist.air ~ d.dist.time.air + I(d.dist.time.air^2))
prd.time.air = data.frame(d.dist.time.air = seq(from =
range(d.dist.time.air)[1], to = range(d.dist.time.air)[2], length.out =
100))
err.time.air = predict(fit.time.air, newdata = prd.time.air, se.fit =
TRUE)

prd.time.air$lci = err.time.air$fit - 1.96 * err.time.air$se.fit
prd.time.air$fit = err.time.air$fit
prd.time.air$uci = err.time.air$fit + 1.96 * err.time.air$se.fit

# fit mantel tests for dist ~ time for air hwy33, air KARE and both
combined
mantel.time.hwy33.air = mantel(d.dist.time.hwy33.air, d.dist.hwy33.air,
method="pearson", permutations=999, parallel = 8)
mantel.time.kare.air = mantel(d.dist.time.kare.air, d.dist.kare.air,
method="pearson", permutations=999, parallel = 8)
mantel.time.air = mantel(d.dist.time.air, d.dist.air, method="pearson",
permutations=999, parallel = 8)

# define points for plotting dist ~ time
df.time.air = data.frame(x = as.numeric(d.dist.time.air), y =
as.numeric(d.dist.air))
df.time.hwy33.air = data.frame(x = as.numeric(d.dist.time.hwy33.air), y =
as.numeric(d.dist.hwy33.air))
df.time.kare.air = data.frame(x = as.numeric(d.dist.time.kare.air), y =
as.numeric(d.dist.kare.air))
```

```
# fit linear models for dist ~ geo for air hwy33, air KARE and both
combined
fit.geo.air.hwy33 = lm(d.dist.hwy33.air ~ d.dist.geo.hwy33.air)
prd.geo.air.hwy33 = data.frame(d.dist.geo.hwy33.air = seq(from =
range(d.dist.geo.hwy33.air)[1], to = range(d.dist.geo.hwy33.air)[2],
length.out = 100))
err.geo.air.hwy33 = predict(fit.geo.air.hwy33, newdata =
prd.geo.air.hwy33, se.fit = TRUE)

prd.geo.air.hwy33$lci = err.geo.air.hwy33$fit - 1.96 *
err.geo.air.hwy33$se.fit
prd.geo.air.hwy33$fit = err.geo.air.hwy33$fit
prd.geo.air.hwy33$uci = err.geo.air.hwy33$fit + 1.96 *
err.geo.air.hwy33$se.fit

fit.geo.air.kare = lm(d.dist.kare.air ~ d.dist.geo.kare.air)
prd.geo.air.kare = data.frame(d.dist.geo.kare.air = seq(from =
range(d.dist.geo.kare.air)[1], to = range(d.dist.geo.kare.air)[2],
length.out = 100))
err.geo.air.kare = predict(fit.geo.air.kare, newdata = prd.geo.air.kare,
se.fit = TRUE)

prd.geo.air.kare$lci = err.geo.air.kare$fit - 1.96 *
err.geo.air.kare$se.fit
prd.geo.air.kare$fit = err.geo.air.kare$fit
prd.geo.air.kare$uci = err.geo.air.kare$fit + 1.96 *
err.geo.air.kare$se.fit

fit.geo.air = lm(d.dist.air ~ d.dist.geo.air)
prd.geo.air = data.frame(d.dist.geo.air = seq(from =
range(d.dist.geo.air)[1], to = range(d.dist.geo.air)[2], length.out =
100))
err.geo.air = predict(fit.geo.air, newdata = prd.geo.air, se.fit = TRUE)

prd.geo.air$lci = err.geo.air$fit - 1.96 * err.geo.air$se.fit
prd.geo.air$fit = err.geo.air$fit
prd.geo.air$uci = err.geo.air$fit + 1.96 * err.geo.air$se.fit

# fit mantel tests for dist ~ geo for air hwy33, air KARE and both
combined
mantel.geo.hwy33.air = mantel(d.dist.geo.hwy33.air, d.dist.hwy33.air,
method="pearson", permutations=999, parallel = 8)
mantel.geo.kare.air = mantel(d.dist.geo.kare.air, d.dist.kare.air,
method="pearson", permutations=999, parallel = 8)
mantel.geo.air = mantel(d.dist.geo.air, d.dist.air, method="pearson",
permutations=999, parallel = 8)

# define points for plotting dist ~ geo
df.geo.air = data.frame(x = as.numeric(d.dist.geo.air), y =
as.numeric(d.dist.air))
df.geo.hwy33.air = data.frame(x = as.numeric(d.dist.geo.hwy33.air), y =
as.numeric(d.dist.hwy33.air))
df.geo.kare.air = data.frame(x = as.numeric(d.dist.geo.kare.air), y =
as.numeric(d.dist.kare.air))
```

```
## test for significant difference between intercepts/slopes between hwy33
and KARE

# time
d.dist.time.hwy33.air.df = data.frame(
        dist =
as.matrix(d.dist.hwy33.air)[lower.tri(as.matrix(d.dist.hwy33.air))],
        time =
as.matrix(d.dist.time.hwy33.air)[lower.tri(as.matrix(d.dist.time.hwy33.air
))],
        site = "hwy33"
)

d.dist.time.kare.air.df = data.frame(
        dist =
as.matrix(d.dist.kare.air)[lower.tri(as.matrix(d.dist.kare.air))],
        time =
as.matrix(d.dist.time.kare.air)[lower.tri(as.matrix(d.dist.time.kare.air))
],
        site = "kare"
)

d.dist.time.air.df = rbind(
        d.dist.time.hwy33.air.df,
        d.dist.time.kare.air.df
)

y = d.dist.time.air.df$dist
x = d.dist.time.air.df$time
site = d.dist.time.air.df$site

fit.time.air.mtest = lm(y ~ site*(x + I(x^2)))
summary(fit.time.air.mtest)

# geo
d.dist.geo.hwy33.air.df = data.frame(
        dist =
as.matrix(d.dist.hwy33.air)[lower.tri(as.matrix(d.dist.hwy33.air))],
        geo =
as.matrix(d.dist.geo.hwy33.air)[lower.tri(as.matrix(d.dist.geo.hwy33.air))
],
        site = "hwy33"
)

d.dist.geo.air.df = data.frame(
        dist = as.matrix(d.dist.air)[lower.tri(as.matrix(d.dist.air))],
        geo =
as.matrix(d.dist.geo.air)[lower.tri(as.matrix(d.dist.geo.air))],
        site = "all"
)

d.dist.geo.air.df = rbind(
        d.dist.geo.hwy33.air.df,
```

```
        d.dist.geo.air.df
)

y = d.dist.geo.air.df$dist
x = d.dist.geo.air.df$geo
site = d.dist.geo.air.df$site

fit.geo.air.mtest = lm(y ~ site*x)
summary(fit.geo.air.mtest)




# soil

# fit linear models for dist ~ time for soil hwy33, soil KARE and both
combined
fit.time.soil.hwy33 = lm(d.dist.hwy33.soil ~ d.dist.time.hwy33.soil)
prd.time.soil.hwy33 = data.frame(d.dist.time.hwy33.soil = seq(from =
range(d.dist.time.hwy33.soil)[1], to = range(d.dist.time.hwy33.soil)[2],
length.out = 100))
err.time.soil.hwy33 = predict(fit.time.soil.hwy33, newdata =
prd.time.soil.hwy33, se.fit = TRUE)

prd.time.soil.hwy33$lci = err.time.soil.hwy33$fit - 1.96 *
err.time.soil.hwy33$se.fit
prd.time.soil.hwy33$fit = err.time.soil.hwy33$fit
prd.time.soil.hwy33$uci = err.time.soil.hwy33$fit + 1.96 *
err.time.soil.hwy33$se.fit

fit.time.soil.kare = lm(d.dist.kare.soil ~ d.dist.time.kare.soil)
#fit.time.soil.kare = lm(d.dist.kare.soil ~ d.dist.time.kare.soil +
I(d.dist.time.kare.soil^2))
prd.time.soil.kare = data.frame(d.dist.time.kare.soil = seq(from =
range(d.dist.time.kare.soil)[1], to = range(d.dist.time.kare.soil)[2],
length.out = 100))
err.time.soil.kare = predict(fit.time.soil.kare, newdata =
prd.time.soil.kare, se.fit = TRUE)

prd.time.soil.kare$lci = err.time.soil.kare$fit - 1.96 *
err.time.soil.kare$se.fit
prd.time.soil.kare$fit = err.time.soil.kare$fit
prd.time.soil.kare$uci = err.time.soil.kare$fit + 1.96 *
err.time.soil.kare$se.fit

fit.time.soil = lm(d.dist.soil ~ d.dist.time.soil)
prd.time.soil = data.frame(d.dist.time.soil = seq(from =
range(d.dist.time.soil)[1], to = range(d.dist.time.soil)[2], length.out =
100))
err.time.soil = predict(fit.time.soil, newdata = prd.time.soil, se.fit =
TRUE)

prd.time.soil$lci = err.time.soil$fit - 1.96 * err.time.soil$se.fit
prd.time.soil$fit = err.time.soil$fit
```

```
prd.time.soil$uci = err.time.soil$fit + 1.96 * err.time.soil$se.fit

# fit mantel tests for dist ~ time for soil hwy33, soil KARE and both
combined
mantel.time.hwy33.soil = mantel(d.dist.time.hwy33.soil, d.dist.hwy33.soil,
method="pearson", permutations=999, parallel = 8)
mantel.time.kare.soil = mantel(d.dist.time.kare.soil, d.dist.kare.soil,
method="pearson", permutations=999, parallel = 8)
mantel.time.soil = mantel(d.dist.time.soil, d.dist.soil, method="pearson",
permutations=999, parallel = 8)

# define points for plotting dist ~ time
df.time.soil = data.frame(x = as.numeric(d.dist.time.soil), y =
as.numeric(d.dist.soil))
df.time.hwy33.soil = data.frame(x = as.numeric(d.dist.time.hwy33.soil), y
= as.numeric(d.dist.hwy33.soil))
df.time.kare.soil = data.frame(x = as.numeric(d.dist.time.kare.soil), y =
as.numeric(d.dist.kare.soil))




# fit linear models for dist ~ geo for soil hwy33, soil KARE and both
combined
fit.geo.soil.hwy33 = lm(d.dist.hwy33.soil ~ d.dist.geo.hwy33.soil)
prd.geo.soil.hwy33 = data.frame(d.dist.geo.hwy33.soil = seq(from =
range(d.dist.geo.hwy33.soil)[1], to = range(d.dist.geo.hwy33.soil)[2],
length.out = 100))
err.geo.soil.hwy33 = predict(fit.geo.soil.hwy33, newdata =
prd.geo.soil.hwy33, se.fit = TRUE)

prd.geo.soil.hwy33$lci = err.geo.soil.hwy33$fit - 1.96 *
err.geo.soil.hwy33$se.fit
prd.geo.soil.hwy33$fit = err.geo.soil.hwy33$fit
prd.geo.soil.hwy33$uci = err.geo.soil.hwy33$fit + 1.96 *
err.geo.soil.hwy33$se.fit

fit.geo.soil.kare = lm(d.dist.kare.soil ~ d.dist.geo.kare.soil)
prd.geo.soil.kare = data.frame(d.dist.geo.kare.soil = seq(from =
range(d.dist.geo.kare.soil)[1], to = range(d.dist.geo.kare.soil)[2],
length.out = 100))
err.geo.soil.kare = predict(fit.geo.soil.kare, newdata =
prd.geo.soil.kare, se.fit = TRUE)

prd.geo.soil.kare$lci = err.geo.soil.kare$fit - 1.96 *
err.geo.soil.kare$se.fit
prd.geo.soil.kare$fit = err.geo.soil.kare$fit
prd.geo.soil.kare$uci = err.geo.soil.kare$fit + 1.96 *
err.geo.soil.kare$se.fit

fit.geo.soil = lm(d.dist.soil ~ d.dist.geo.soil)
```

```
prd.geo.soil = data.frame(d.dist.geo.soil = seq(from =
range(d.dist.geo.soil)[1], to = range(d.dist.geo.soil)[2], length.out =
100))
err.geo.soil = predict(fit.geo.soil, newdata = prd.geo.soil, se.fit =
TRUE)

prd.geo.soil$lci = err.geo.soil$fit - 1.96 * err.geo.soil$se.fit
prd.geo.soil$fit = err.geo.soil$fit
prd.geo.soil$uci = err.geo.soil$fit + 1.96 * err.geo.soil$se.fit

# fit mantel tests for dist ~ geo for soil hwy33, soil KARE and both
combined
mantel.geo.hwy33.soil = mantel(d.dist.geo.hwy33.soil, d.dist.hwy33.soil,
method="pearson", permutations=999, parallel = 8)
mantel.geo.kare.soil = mantel(d.dist.geo.kare.soil, d.dist.kare.soil,
method="pearson", permutations=999, parallel = 8)
mantel.geo.soil = mantel(d.dist.geo.soil, d.dist.soil, method="pearson",
permutations=999, parallel = 8)

# define points for plotting dist ~ geo
df.geo.soil = data.frame(x = as.numeric(d.dist.geo.soil), y =
as.numeric(d.dist.soil))
df.geo.hwy33.soil = data.frame(x = as.numeric(d.dist.geo.hwy33.soil), y =
as.numeric(d.dist.hwy33.soil))
df.geo.kare.soil = data.frame(x = as.numeric(d.dist.geo.kare.soil), y =
as.numeric(d.dist.kare.soil))

# test for significant difference between intercepts/slopes between hwy33
and KARE

# time
d.dist.time.hwy33.soil.df = data.frame(
        dist =
as.matrix(d.dist.hwy33.soil)[lower.tri(as.matrix(d.dist.hwy33.soil))],
        time =
as.matrix(d.dist.time.hwy33.soil)[lower.tri(as.matrix(d.dist.time.hwy33.so
il))],
        site = "hwy33"
)

d.dist.time.kare.soil.df = data.frame(
        dist =
as.matrix(d.dist.kare.soil)[lower.tri(as.matrix(d.dist.kare.soil))],
        time =
as.matrix(d.dist.time.kare.soil)[lower.tri(as.matrix(d.dist.time.kare.soil
))],
        site = "kare"
)

d.dist.time.soil.df = rbind(
        d.dist.time.hwy33.soil.df,
        d.dist.time.kare.soil.df
)
```

```
y = d.dist.time.soil.df$dist
x = d.dist.time.soil.df$time
site = d.dist.time.soil.df$site

fit.time.soil.mtest = lm(y ~ site*(x))
summary(fit.time.soil.mtest)

# geo
d.dist.geo.hwy33.soil.df = data.frame(
        dist = 
as.matrix(d.dist.hwy33.soil)[lower.tri(as.matrix(d.dist.hwy33.soil))],
        geo = 
as.matrix(d.dist.geo.hwy33.soil)[lower.tri(as.matrix(d.dist.geo.hwy33.soil
))],
        site = "hwy33"
)

d.dist.geo.soil.df = data.frame(
        dist = as.matrix(d.dist.soil)[lower.tri(as.matrix(d.dist.soil))],
        geo = 
as.matrix(d.dist.geo.soil)[lower.tri(as.matrix(d.dist.geo.soil))],
        site = "all"
)

d.dist.geo.soil.df = rbind(
        d.dist.geo.hwy33.soil.df,
        d.dist.geo.soil.df
)

y = d.dist.geo.soil.df$dist
x = d.dist.geo.soil.df$geo
site = d.dist.geo.soil.df$site

fit.geo.soil.mtest = lm(y ~ site*x)
summary(fit.geo.soil.mtest)

# combine and export mtest results
fit.mtest.combined = rbind(
        summary(fit.time.air.mtest)$coefficients,
        summary(fit.geo.air.mtest)$coefficients,
        summary(fit.time.soil.mtest)$coefficients,
        summary(fit.geo.soil.mtest)$coefficients
)
fit.mtest.combined = round(fit.mtest.combined, 4)

# compare slopes for geographic distance decay between hwy33 air and all 
air
# (see if land use is a significant interaction term)

# Make lm and anova table with "site" as interaction term
m.interaction = lm(dist ~ geo*site, data = d.dist.geo.air.df)
anova(m.interaction)

# Obtain slopes
```

```
m.interaction$coefficients
m.lst <- lstrends(m.interaction, "site", var="geo")

# Compare slopes
pairs(m.lst)

# compare slopes for temporal distance decay between hwy33 air and kare
air (parabola)
# (see if land use is a significant interaction term)

# Make lm and anova table with "site" as interaction term
m.interaction = lm(dist ~ time*site + I(time^2)*site, data =
d.dist.time.air.df)
anova(m.interaction)

# Obtain slopes
m.interaction$coefficients
m.lst <- lstrends(m.interaction, "site", var="geo")

# Compare slopes
pairs(m.lst)




### Linear Mixed Effects Model (Onygenales Abundance)

# load libraries
library(lme4)
library(lmerTest)
library(multcomp)
library(lsmeans)
library(pbkrtest)
library(MuMIn)
library(vegan)

# load data
d = read.csv("d.combined.genus.onygenales.csv")
d.meta = d[,1:9]

# add factor for sitetype
d.meta$sitetype = paste(d.meta$site, d.meta$type, sep = " ")

# add factor for sitetype
d.meta$sitetype = paste(d.meta$site, d.meta$type, sep = " ")

# extract numeric data for number wrangling
d.numeric = d[,10:(ncol(d))]

# sum total abundance for each sample
total.abundance = rowSums(d.numeric)

# make new dataframe
```

```
d.total = d.meta
d.total$Onygenales = rowSums(d.numeric)

# define variables
# note: Site = "Land Use", site = "Site", year = "Year", month = "Month",
type = "Medium"
y = d.total$Onygenales
month= d.total$month
sitetype = d.total$sitetype

# fit model with varying intercepts for month and type and fixed effect
for site
lmer_fit_REML = lmer(y ~ sitetype + (1|month), REML=T)

# extract coefficients
coefs = round(data.frame(coef(summary(lmer_fit_REML))), 3)
coefs

# fit alternative model
lmer_fit = lmer(y ~ sitetype + (1|month), REML=F)

# fit null model
lmer_fit_null = lmer(y ~ (1|month), REML=F)

# compare models using log-likelihood ratio
anova(lmer_fit, lmer_fit_null)


# multiple comparison with glht (z-statistic, less conservative)
summary(glht(lmer_fit_REML,mcp(sitetype="Tukey")))

# multiple comparison with lsmeans (t-statistic, more conservative,
kenwood-rogers)
lsmeans(lmer_fit_REML, pairwise ~ sitetype)





### Linear Mixed Effects Model (Plant Pathogen Guild)

# load libraries
library(lme4)
library(lmerTest)
library(multcomp)
library(lsmeans)
library(pbkrtest)
library(MuMIn)
library(vegan)
library(reshape2)

# load data
d = read.csv("d.combined.guilds.csv")
```

```
# remove rows containing no guilds
d = d[which(rowSums(d[,9:dim(d)[2]]) != 0),]

d.matrix = as.matrix(d[,9:dim(d)[2]])

# remove any guilds not representing at least 1% of the community across
all samples
d.matrix = d.matrix[,(colSums(d.matrix)/sum(colSums(d.matrix))) >= .01]

# convert data to percent abundance
d.matrix = prop.table(d.matrix, 1)

# load meta data
d.meta = d[,1:8]

# standardize date column
d.meta$date = strptime((paste(d.meta$day, d.meta$month, d.meta$year)),
format = "%e %B %Y")

# make numeric month column
d.meta$month.numeric = format(d.meta$date, "%m")


# aggregate data
d.aggregate = aggregate(d.matrix, by = list(d.meta$month, d.meta$site,
d.meta$type), mean)
names(d.aggregate)[1:3] = c("month", "site", "type")

# change factor names for plotting
d.aggregate[d.aggregate$site!="kare",]$site = paste("Hwy33-",
d.aggregate[d.aggregate$site!="kare",]$site, sep = "")
d.aggregate[d.aggregate$site=="kare",]$site = "KARE"
d.aggregate[d.aggregate$type=="air",]$type = "Air"
d.aggregate[d.aggregate$type=="soil",]$type = "Soil"

# melt data
d.melt = melt(d.aggregate, id.vars = list("month", "site", "type"))

# add numeric month vector
d.melt$month.numeric = as.factor(match(d.melt$month, month.name))

# add abbreviated month vector
d.melt$month.abv = factor(substr(d.melt$month, 1, 3), levels =
substr(month.name, 1, 3))

# replace . with space in guild names for clearer visualization
d.melt$variable = gsub("\\.", " ", d.melt$variable)

# make air only dataframe
d.melt.air = d.melt[which(d.melt$type=="Air"),]

# reorder months so nov and dec are 1st
d.melt.air$month.numeric = as.numeric(d.melt.air$month.numeric)
```

```
d.melt.air$month.numeric = d.melt.air$month.numeric + 2
d.melt.air[which(d.melt.air$month.numeric == 13),]$month.numeric = 1
d.melt.air[which(d.melt.air$month.numeric == 14),]$month.numeric = 2

# make plant pathogen only graph
d.melt.air.plant = d.melt.air[which(d.melt.air$variable=="Plant
Pathogen"),]

# make plant pathogen only graph with only may - oct
d.melt.air.plant.summer = d.melt.air.plant[which(d.melt.air.plant$month
%in% month.name[5:10]),]

# adjust d.meta
# reorder months so nov and dec are 1st
d.meta$month.numeric = as.numeric(d.meta$month.numeric)
d.meta$month.numeric = d.meta$month.numeric + 2
d.meta[which(d.meta$month.numeric == 13),]$month.numeric = 1
d.meta[which(d.meta$month.numeric == 14),]$month.numeric = 2

# make air only matrix
d.matrix.air = d.matrix[which(d.meta$type=="air"),]

# make air only meta
d.meta.air = d.meta[which(d.meta$type=="air"),]

# change factor names for plotting (d.meta.air)
d.meta.air[d.meta.air$site!="kare",]$site = paste("Hwy33-",
d.meta.air[d.meta.air$site!="kare",]$site, sep = "")
d.meta.air[d.meta.air$site=="kare",]$site = "KARE"
d.meta.air[d.meta.air$type=="air",]$type = "Air"

# make air only matrix (summer)
d.matrix.air.summer= d.matrix.air[which(d.meta.air$month %in%
month.name[5:10]),]

# make air only meta (summer)
d.meta.air.summer= d.meta.air[which(d.meta.air$month %in%
month.name[5:10]),]

# statistics

# define variables
y = d.matrix.air.summer[,4]
month= d.meta.air.summer$month.numeric
site = as.factor(d.meta.air.summer$site)

# fit linear model
lm_fit = lm(y ~ month + site)
summary(lm_fit)

# fit model with varying intercepts for month and type and fixed effect
for site
lmer_fit_REML = lmer(y ~ month + (1|site), REML=T)
```

```
# extract coefficients
coefs = round(data.frame(coef(summary(lmer_fit_REML))), 3)
coefs

# fit alternative model
lmer_fit = lmer(y ~ month + (1|site), REML=F)

# fit null model
lmer_fit_null = lmer(y ~ (1|site), REML=F)

# compare models using log-likelihood ratio
model_compare_1 = anova(lmer_fit, lmer_fit_null)
model_compare_1

# extract p, marginal and conditional R^2 values
p = round(model_compare_1[2,8], 3)
r2m = round(r.squaredGLMM(lmer_fit)[1], 3)
r2c = round(r.squaredGLMM(lmer_fit)[2], 3)

#make labels for p and r^2 values
rp0 = as.expression(" " ~ " " ~ " ")
rp1 = as.expression(bquote({r^{2}}[m] ~ "=" ~ .(format(r2m, digits = 2))))
rp2 = as.expression(bquote({r^{2}}[c] ~ "=" ~ .(format(r2c, digits = 2))))
#rp3 = as.expression(bquote(p[lme] ~ "=" ~ .(format(p, digits = 2))))
rp3 = as.expression(bquote(p[lme] ~ "<" ~ .(format(0.001, digits = 2))))
rp = c(rp3, rp2, rp1)

# multiple comparison with glht (z-statistic, less conservative)
multiple_glht = summary(glht(lmer_fit_REML,mcp(site="Tukey")))
multiple_glht[1]

# multiple comparison with lsmeans (t-statistic, more conservative,
kenwood-rogers)
multiple_lsmeans = as.data.frame(lsmeans(lmer_fit_REML,
pairwise~site)$contrasts)
multiple_lsmeans[,2:6] = apply(multiple_lsmeans[,2:6], 2, function (x)
{round(x, 3)})
colnames(multiple_lsmeans) = c("", "Estimate", "se", "df", "t", "p")
rownames(multiple_lsmeans) = multiple_lsmeans[,1]
multiple_lsmeans = multiple_lsmeans[,-c(1,3)]
multiple_lsmeans$p[c(1,3,4,6)] = "<0.001"
multiple_lsmeans
```
