# Supplementary material for: The air mycobiome is decoupled from the soil mycobiome in the California San Joaquin Valley
Source: Mol Ecol. 2022 Aug 25;31(19):4962–78. doi: 10.1111/mec.16640 (PMC9624177; doi:10.1111/mec.16640)
Supplement: Supplementary file 2 — Appendix S2 [file MEC-31-4962-s001.pdf]

MOLECULAR ECOLOGY

Supplemental Information for:

The air mycobiome is decoupled from the soil mycobiome in the California San Joaquin Valley

Robert Wagner, Liliam Montoya, Cheng Gao, Jennifer R. Head, Justin Remais, John W. Taylor

Table of Contents:

Figure S1. Sampler photograph	Page 2
Figure S2. Species richness	Page 3
Figure S3. Species richness	Page 4
Figure S4. Venn diagram	Page 5
Figure S5. Principal coordinate	Page 6
Figure S6. Principal coordinate	Page 7
Figure S7. Principal coordinate	Page 8
Figure S8. Principal coordinate	Page 9
Figure S9. KARE, years	Page 10
Figure S10. Plant Pathogens	Page 11
Figure S11. Top 10 phyla	Page 12
Figure S12. Top 30 Orders	Page 13
Figure S13. Onygenales genera	Page 14
Figure S14. Sequencing depth	Page 15
Table S1. Previous Publications	Page 16
Table S2. Site Coordinates	Page 17
Table S3. Species richness	Page 18
Table S4. Species richness	Page 19
Table S5. Pairwise PERMANOVA	Page 20
Table S6. PERMANOVA	Page 21
Table S7. PERMANOVA	Page 22
References	Page 23

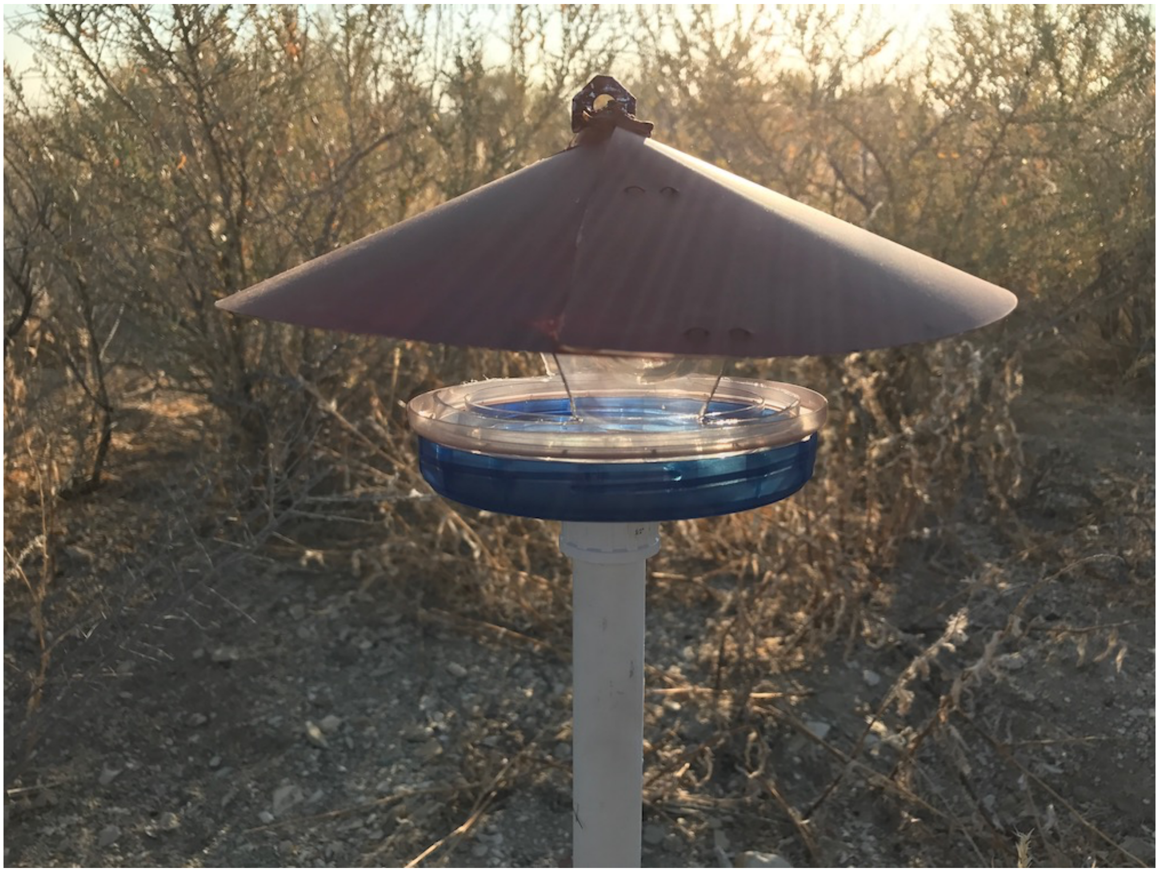

Figure S1. Photograph of settled dust sampler used to sample the air mycobiome in the San Joaquin Valley. The sampler rests atop a polyvinylchloride pipe that is slipped over and secured to a reinforcing rod driven into the ground so that the sampler is 50cm above the soil surface. A plastic cone was affixed to the top of the sampler to prevent precipitation from impacting the open petri dish within. The sides of the sampler were open to ambient air to allow dust carried on air currents to passively settle on the petri dish. Vertical deposition during periods of still air may have been inhibited.

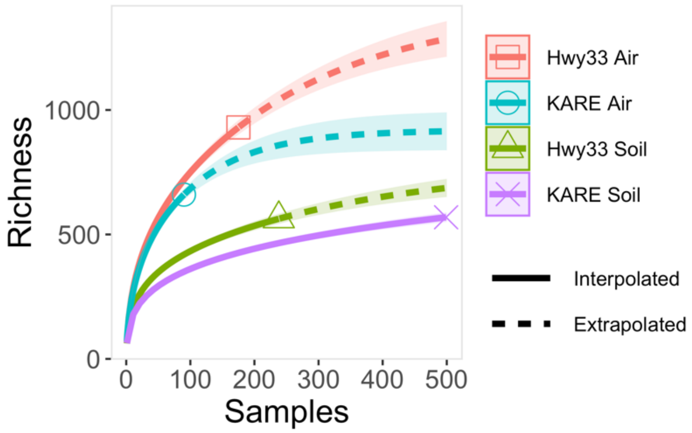

Figure S2. Species richness as a function of sampling effort across all sites. Points and interpolated lines represent actual sampling effort. Extrapolated lines estimate species richness at higher potential sampling efforts. Shaded regions = 95% confidence interval derived from a bootstrap estimate of variance with 1000 replications.

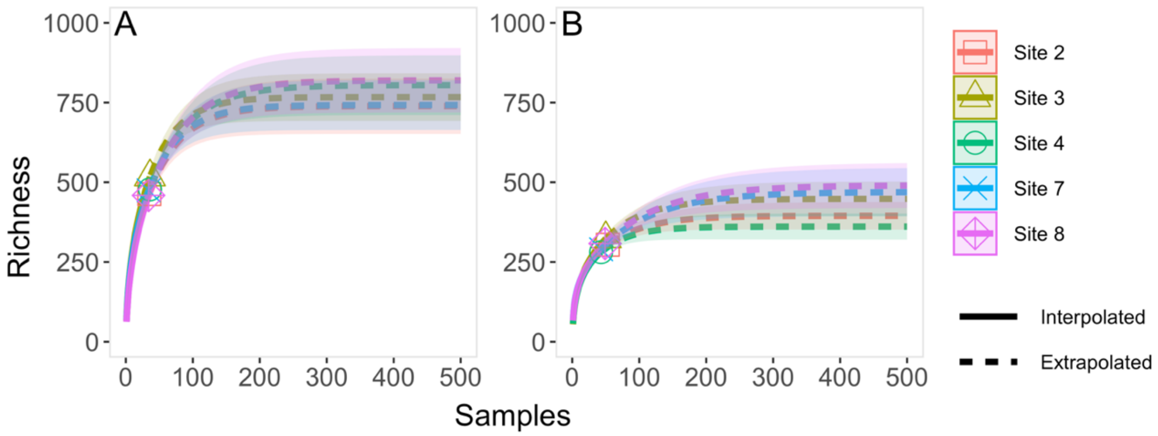

Figure S3. Species richness as a function of sampling effort at Hwy33 sites for air and settled dust samplers (A) and rodent burrow soils (B). Points and interpolated lines represent actual sampling effort. Extrapolated lines estimate species richness at higher potential sampling efforts. Shaded regions = 95% confidence interval derived from a bootstrap estimate of variance with 1000 replications.

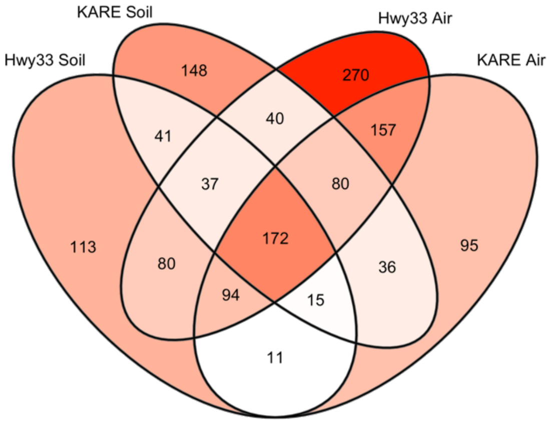

Figure S4. Venn diagram showing the number of species unique to, and shared between, each land use type (Hwy33 vs KARE) and sampling medium (soil vs air) combination.

A B

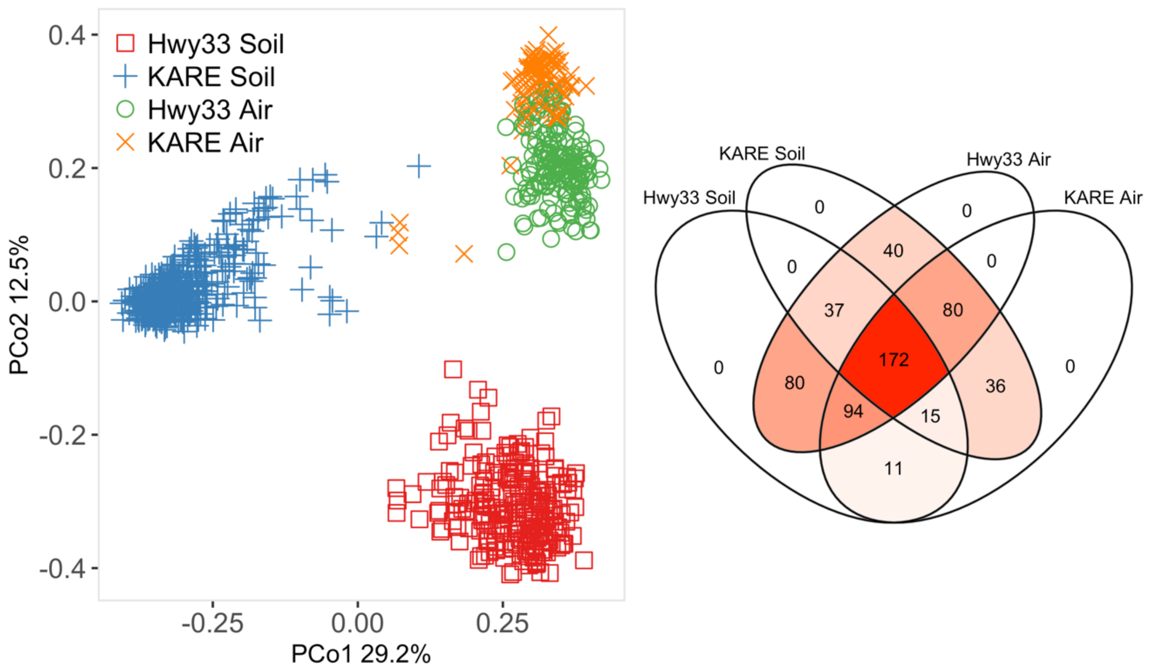

C

	Df	SumOfSqs	r^2	F	p
Land Use	1	69.9	0.21	497.54	0.001
Site	4	7.56	0.02	13.46	0.001
Year	1	11.43	0.03	81.37	0.001
Month	11	17.69	0.05	11.44	0.001
Medium	1	40.67	0.12	289.45	0.001
Land Use : Medium	1	15.8	0.05	112.46	0.001
Land Use : Site : Medium	4	4.16	0.01	7.4	0.001
Land Use : Site : Year : Medium	10	4.69	0.01	3.34	0.001
Land Use : Site : Year : Month : Medium	104	36.78	0.11	2.52	0.001
Residual	863	121.25	0.37		
Total	1000	329.93	1		

Figure S5. Principal coordinate analysis limited to species found in both air and soil samples, which separates into the same three groups as the full dataset: agricultural (KARE) soil, undeveloped (Hwy33) soil, and air from both agricultural and undeveloped land (KARE and Hwy33) (A). Venn diagram showing the number of species unique to, and shared between, each land-use and sampling medium combination (B). Nested PERMANOVA coefficient table (C).

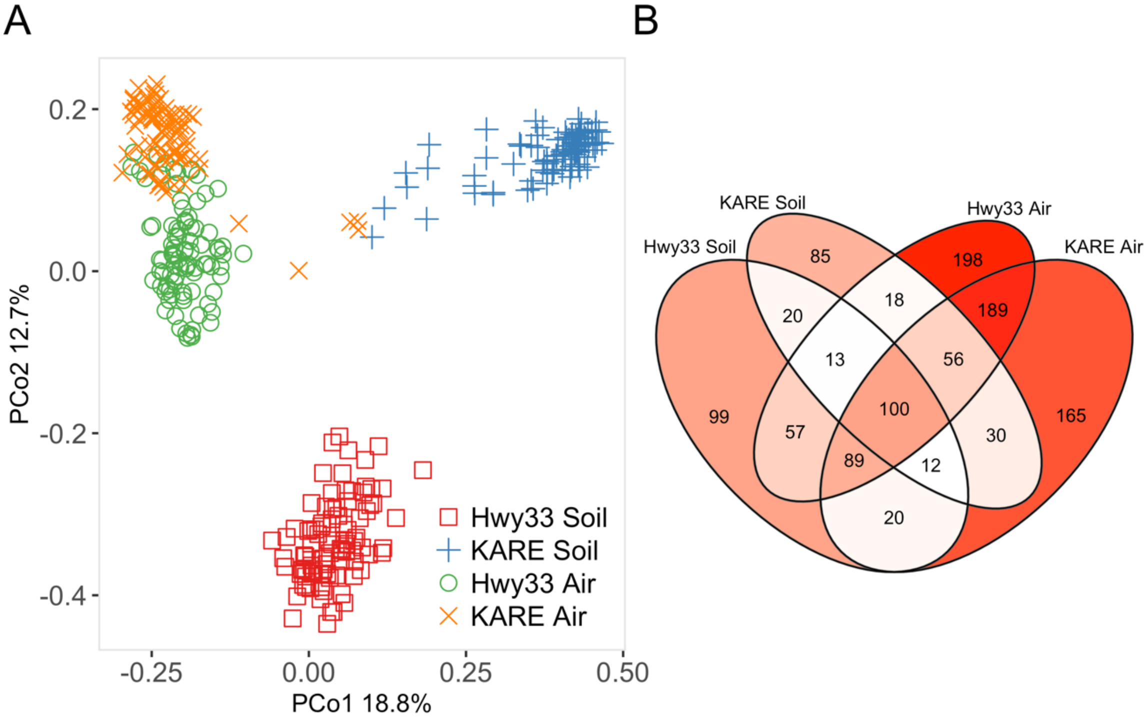

C

	Df	SumOfSqs	r^2	F	p
Land Use	1	11.82	0.1	73.25	0.001
Site	4	3.23	0.03	5	0.001
Year	1	6.57	0.06	40.72	0.001
Month	11	10.3	0.09	5.8	0.001
Medium	1	13.3	0.11	82.38	0.001
Land Use : Medium	1	6.03	0.05	37.36	0.001
Land Use : Site : Medium	4	2.22	0.02	3.44	0.001
Land Use : Site : Year : Medium	10	3.61	0.03	2.24	0.001
Land Use : Site : Year : Month : Medium	82	22.37	0.19	1.69	0.001
Residual	244	39.39	0.33		
Total	359	118.85	1		

Figure S6. Principal coordinate analysis after balancing the number of samples from the four combinations of land-use and sampling medium (Hwy33 Soil, Hwy33 Air, KARE Soil, KARE Air) by randomly selecting 90 samples from the abundantly sampled groups to match the lower number from KARE Air. (A). Venn diagram showing the number of species unique to, and shared between, each land-use and sampling medium combination (B). Nested PERMANOVA coefficient table (C).

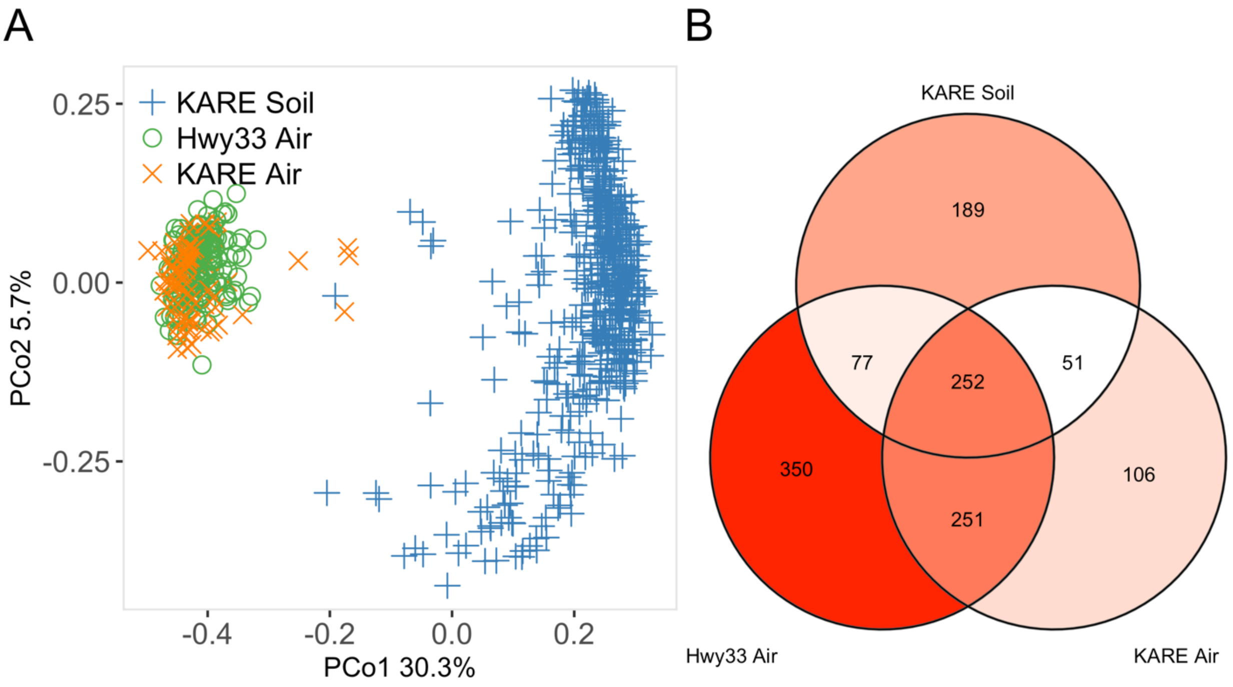

C

	Df	SumOfSqs	r^2	F	p
Land Use	1	41.7	0.17	253.85	0.001
Site	4	2.51	0.01	3.81	0.001
Year	1	12.78	0.05	77.8	0.001
Month	11	19.82	0.08	10.97	0.001
Medium	1	25.81	0.11	157.11	0.001
Land Use : Site : Year : Medium	5	2.81	0.01	3.43	0.001
Land Use : Site : Year : Month : Medium	54	23.96	0.1	2.7	0.001
Residual	686	112.69	0.47		
Total	763	242.08	1		

Figure S7. Principal coordinate analysis after removing all Hwy33 soil samples from the analysis, leaving only three combinations of land-use and sampling medium (KARE Soil, Hwy33 Air and KARE Air) (A). Venn diagram showing the number of species unique to, and shared between, each land-use and sampling medium combination (B). Nested PERMANOVA coefficient table (C).

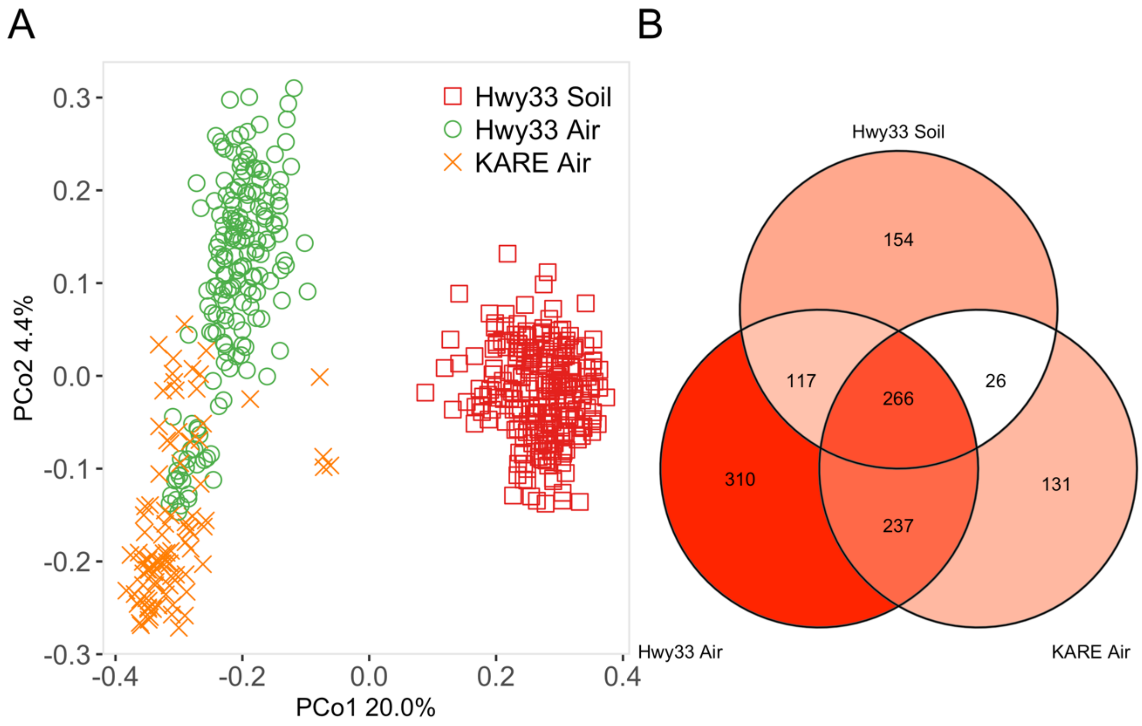

C

	Df	SumOfSqs	r^2	F	p
Land Use	1	13.9	0.08	70.19	0.001
Site	4	7.79	0.04	9.84	0.001
Year	1	2.27	0.01	11.48	0.001
Month	11	10.63	0.06	4.88	0.001
Medium	1	24.12	0.14	121.81	0.001
Land Use : Site : Medium	4	4.71	0.03	5.94	0.001
Land Use : Site : Year : Medium	9	3.53	0.02	1.98	0.001
Land Use : Site : Year : Month : Medium	95	33.98	0.19	1.81	0.001
Residual	376	74.47	0.42		
Total	502	175.39	1		

Figure S8. Principal coordinate analysis after removing all KARE soil samples from the analysis, leaving only three combinations of land-use and sampling medium (Hwy33 Soil, Hwy33 Air and KARE Air) (A). Venn diagram showing the number of species unique to, and shared between, each land-use and sampling medium combination (B). Nested PERMANOVA coefficient table (C).

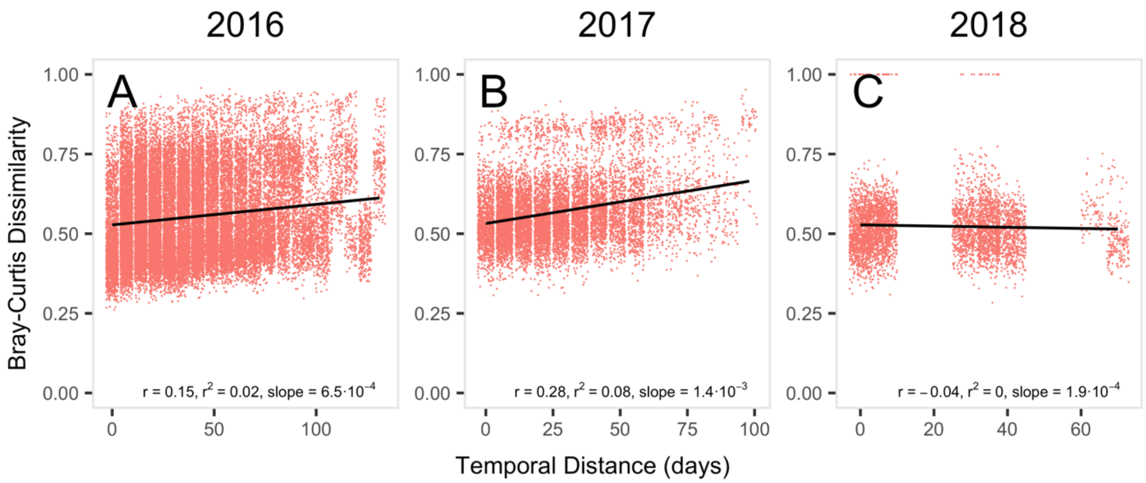

Figure S9. Bray-Curtis dissimilarity as a function of temporal distance (days) at KARE in 2016 (A), 2017 (B) and 2018 (C). Significant relationships were present in 2016 and 2017 but not in 2018. Mantel $p = 0.001$ in 2016 and 2017. Mantel $p = 0.7$ in 2018. $n = 254$ in 2016, 147 in 2017 and 98 in 2018. r = Mantel statistic. r^2 = linear model coefficient of determination. Slopes differed significantly between all pairs of years ($p < 0.001$). Points jittered up to ±3 units on the x-axis for visibility. Note: x-axis range differs between panels.

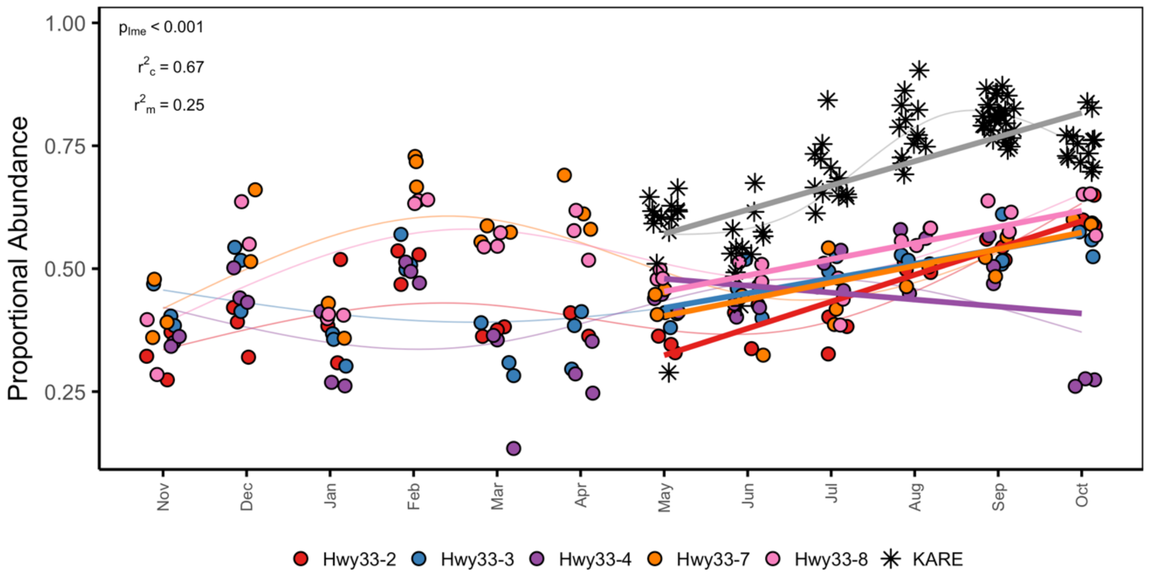

Figure S10. Proportional abundance of airborne taxa assigned to the Plant Pathogen functional guild as a function of month and site. For settled dust samples collected from May through October (n = 180), a linear mixed effects model was calculated with month as a fixed effect and site as a random effect. P_{lme} = p-value obtained from log-likelihood test between full model (site and month) and null model (excluding month). r^2_c = conditional r^2 (fixed effect [month] + random effect [site]). r^2_m = marginal r^2 (only random effect [site]). Thick lines = linear regression lines for each site from May through October. Thin lines = natural cubic spline regressions (3 degrees of freedom) for each site. Note: x-axis is from November 2017 through October 2018. Points represent individual settled dust samples and are jittered on the x-axis for clarity.

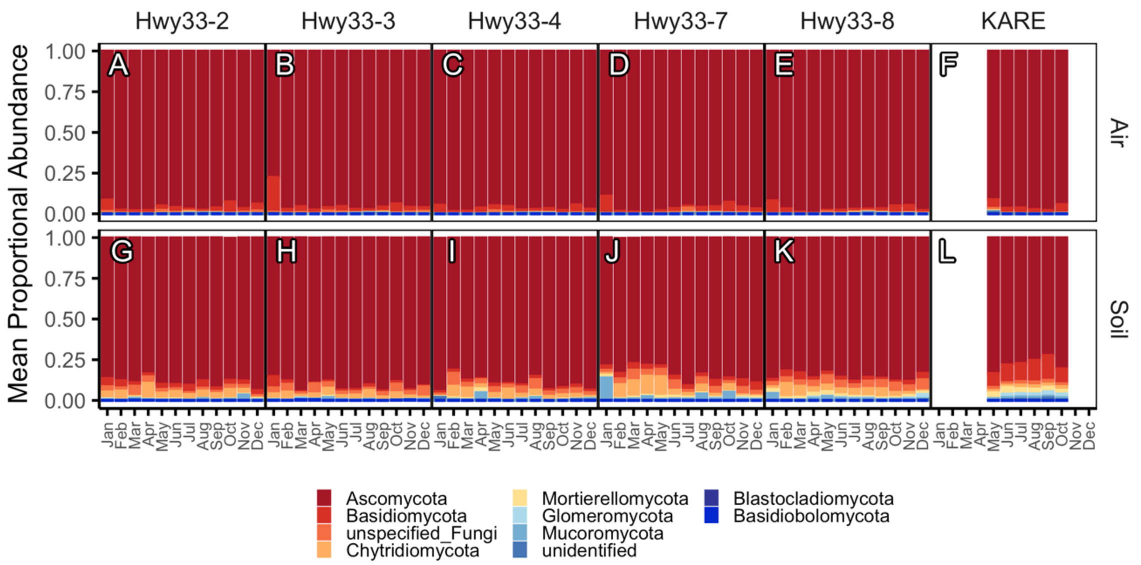

Figure S11. Mean proportional abundance of the top 10 most abundant phyla, among all phyla, as a function of month, site and sampling medium. Values are means between replicates, and across years (for KARE samples). unidentified = all pooled phyla matching unidentified reference sequences. unspecified = sequences binned into a taxonomic level without a reference sequence. Note that November and December (2017) precede January – October (2018) for Hwy33.

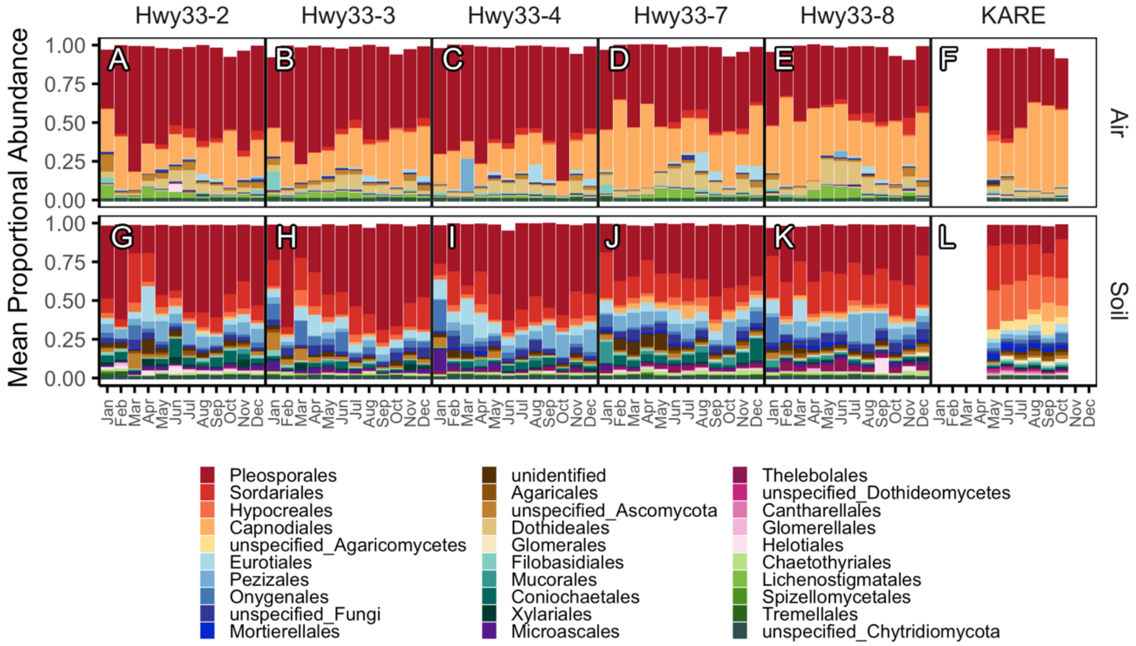

Figure S12. Mean proportional abundance of the top 30 most abundant orders, among all orders, as a function of month, site and sampling medium from ITS2 sequences. Values are means between replicates, and across years (for KARE samples). unidentified = all pooled orders matching unidentified reference sequences. unspecified = sequences binned into a taxonomic level without a reference sequence. Note that November and December (2017) precede January – October (2018) for Hwy33.

A

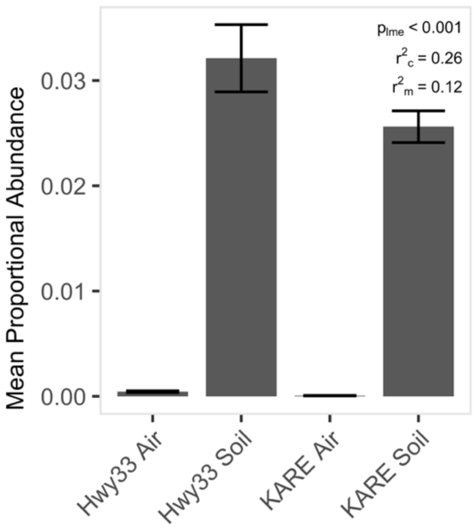

B

	mean	sd	sem	n
Hwy33 Air	0.00044	0.00116	0.00009	175
Hwy33 Soil	0.03211	0.04924	0.00319	238
KARE Air	0.00006	0.00016	0.00002	90
KARE Soil	0.02561	0.03373	0.00151	500

C

	Estimate	df	t	p
Hwy33 Air - Hwy33 Soil	−0.033	993.577	−10.064	<0.001
Hwy33 Air - KARE Air	−0.007	996.572	−1.589	0.385
Hwy33 Air - KARE Soil	−0.032	959.467	−9.387	<0.001
Hwy33 Soil - KARE Air	0.026	993.843	5.957	<0.001
Hwy33 Soil - KARE Soil	0.001	935.139	0.449	0.97
KARE Air - KARE Soil	−0.025	992.468	−6.407	<0.001

Figure S13. Mean proportional abundance of Onygenales genera as a function of land use (Hwy33 vs KARE) and sampling medium (soil vs air) (A, B). A linear mixed effects model was calculated with a factor combining land use and sampling medium "site-medium" (Hwy33 Air, KARE Soil, etc.) as a fixed effect and sampling month as a random effect. P_{lme} = p-value obtained from log-likelihood test between full model (site-medium and month) and null model (excluding site-medium). r^2_c = conditional r^2 (fixed effect[site-medium] + random effect [month]). r^2_m = marginal r^2 (only random effect [month]). Error bars = SEM. Pairwise comparison of individual factor levels obtained from the mixed effects model (C).

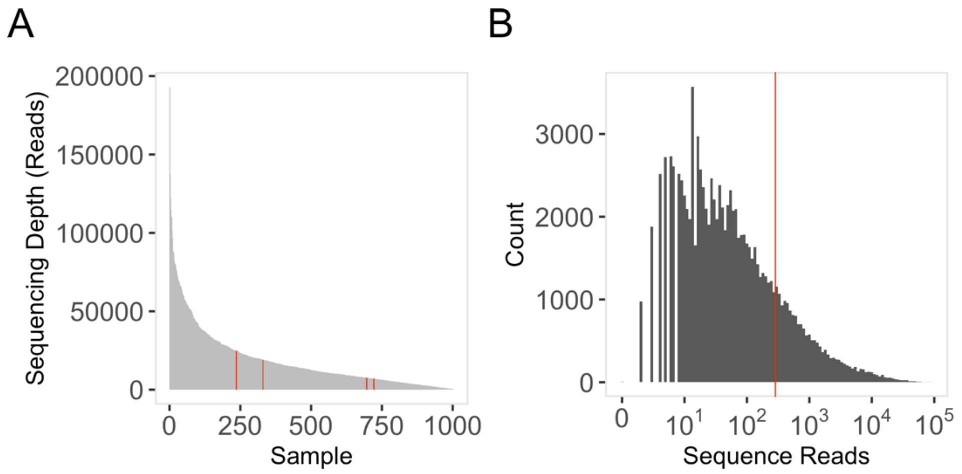

Figure S14. Sequencing depth as a function of sample, ordered from highest to lowest, with samples where *Coccidioides* was detected in red (A). Distribution of total sequence reads assigned to each species across all samples on a logarithmic scale. Vertical red bar indicates the position of *Coccidioides* reads in the distribution (B).

Table S1. The current study (yellow, bold text) and publications investigating the outdoor air mycobiome *with* the soil mycobiome (tan) and the outdoor air mycobiome *without* the soil mycobiome (blue) using high-throughput sequencing methods. In some cases, elevation and sampler height values were estimated based on methods and site descriptions. This list is extensive, though not necessarily exhaustive.

Study	Air Samples	Soil Samples	Location	Sampling Method	Elevation	Sampler Height	DNA Region	Sequencer
**Current Study**	**265**	**737**	**California**	**Deposition**	**103m - 361m**	**0.5m**	**ITS2**	**Illumina Miseq**
(Schiro et al., 2022)*	12	87	Arizona	Impaction	600m - 1400m	0m - 0.05m	ITS1	Illumina Miseq
(Abrego et al., 2020)	90	90	Finland	Impaction	7m - 100m	0m	ITS2	Illumina Miseq
(Abrego et al., 2018)	134	35	Finland	Impaction	0m - 126m	0m - 10m	ITS1, ITS2	Roche 454
(Kivlin et al., 2014)	25 - 40	63	California	Filtration	520m - 1680m	7m	18s	Roche 454
(Redondo et al., 2022)	322	-	Sweden	Deposition	15m - 50m	1.5m	ITS2	PacBio SMRT
(Niu et al., 2021)	11	-	Tianjin	Impaction	6m	21m	ITS1	Illumina HiSeq
(Sánchez-Parra et al., 2021)	15	-	Spain	Impaction	1000m	1.5m - 1000m	ITS1, ITS2	Illumina Miseq
(Redondo et al., 2020)	1157	-	Sweden	Deposition, Impaction	50m - 110m	1m - 8m	ITS2	PacBio SMRT
(Núñez & Moreno, 2020)	8	-	Spain	Impaction	640m	80m - 250m	ITS1, ITS2	Illumina Miseq
(Tipton et al., 2019)	383	-	Hawaii	Filtration	3397m	Unknown	ITS1	Illumina Miseq
(Tignat-Perrier et al., 2019)	75	-	Global	Filtration	Variable	Unknown	ITS2	Illumina Miseq
(Du et al., 2018)	104	-	Beijing	Filtration	88m	30m	ITS1	Illumina Miseq
(Chen et al., 2018)	98	-	Canada	Deposition, Impaction	20 - 60m	0m - 1.2m	ITS1, ITS2	Roche 454
(Cáliz et al., 2018)	150	-	Spain	Deposition, Filtration	1800m	Unknown	18s	Illumina Miseq
(Woo et al., 2018)	58	-	South Korea	Deposition, Filtration	109m	20m	ITS1	Illumina Miseq
(Castaño et al., 2017)	64	-	Spain	Deposition	670m	30cm	ITS2	Illumina Miseq
(Nicolaisen et al., 2017)	193	-	Europe	Impaction	9m - 130m	10m - 15m	ITS1	Roche 454
(Yan et al., 2016)	81	-	Beijing	Impaction	51m	8m	ITS1	Illumina Miseq
(Barberán et al., 2015)	1289	-	United States	Deposition	Variable	2m - Unknown	ITS1	Illumina Miseq, HiSeq
(Womack et al., 2015)	4	-	Amazonia	Impaction	67m	48m	D1/D2 LSU	Illumina Miseq, HiSeq
(Peay & Bruns, 2014)	178	-	California	Deposition	64m	Unknown	ITS1, ITS2	Roche 454
(Adams et al., 2013)	84	-	United States	Deposition	Unknown	Unknown	ITS1	Roche 454
(Yamamoto et al., 2012)	20	-	Connecticut	Filtration	12m	22m	ITS1, ITS2	Roche 454
(Fröhlich-Nowoisky et al., 2012)	136	-	Global	Variable	Variable	Variable	ITS1, ITS2	ABI Prism 3xxx
(Frohlich-Nowoisky et al., 2009)	42	-	Germany	Filtration, Impaction	127m	16m	ITS1, 18s	ABI Prism 3xxx
(Bowers et al., 2009)	11	-	Colorado	Filtration	3200m	4m	18s	ABI Prism 3xxx
(Fierer et al., 2008)	5	-	Colorado	Impaction	1660m	1.5m	18s	ABI Prism 3730

*Sampling was from dust generated by artificially disturbing the soil surface.

Table S2. Site latitude and longitude in decimal degrees and distance from California Highway 33 (Hwy33 sites).

Site	Latitude	Longitude	Distance from highway (Hwy33)
Hwy33 2	35.176195	-119.52282	56m
Hwy33 3	35.339717	-119.63083	90m
Hwy33 4	35.474297	-119.72277	147m
Hwy33 7	35.570672	-119.82144	515m
Hwy33 8	35.795795	-119.98771	80m
KARE	36.600289	-119.51099	NA

Table S3. Species richness as a function of sampling effort. Richness estimates were calculated in iNEXT at 500 samples. 95% confidence interval derived from a bootstrap estimate of variance with 1000 replications.

Site	Observed	Estimated	Std. Error	Lower 95% CI	Upper 95% CI
Hwy33 Air	930	1409.53	56.65	1298.5	1520.56
KARE Air	660	918.97	35.97	848.47	989.48
Hwy33 Soil	563	779.97	36.41	708.6	851.34
KARE Soil	569	759.3	34.37	691.93	826.66

Table S4. Species richness as a function of sampling effort for Hwy33 samples. Richness estimates were calculated in iNEXT at 500 samples. 95% confidence interval derived from a bootstrap estimate of variance with 1000 replications.

Medium	Site	Observed	Estimated	Std. Error	Lower 95% CI	Upper 95% CI
Air	Site 2	463	738.84	41.69	657.13	820.55
Air	Site 3	517	767.13	38.00	692.66	841.61
Air	Site 4	478	804.67	50.11	706.46	902.89
Air	Site 7	475	742.44	42.95	658.26	826.62
Air	Site 8	459	819.94	50.26	721.43	918.45
Soil	Site 2	304	394.88	22.47	350.85	438.91
Soil	Site 3	323	448.13	27.31	394.60	501.66
Soil	Site 4	281	361.11	21.44	319.09	403.12
Soil	Site 7	290	469.80	37.60	396.11	543.49
Soil	Site 8	308	490.68	36.97	418.21	563.14

Table S5. Pairwise PERMANOVA coefficient table for the Bray-Curtis dissimilarity among samples as a function of a factor combining land use and sampling medium (pairwiseadonis function). Permutations = 1000 (unstratified). n = 1002. F = pseudo F-ratio (Anderson, 2001). Note: very low p-values are likely a result of greatly increased sensitivity due to high replication (van der Laan et al., 2010), whereas r^2 and F values can differentiate between important and trivial independent variables.

	Sum of Squares	F	r^2	p value	Adjusted p value
KARE Soil vs Hwy33 Soil	65.28	310.8	0.30	0.001	0.006
KARE Soil vs Hwy33 Air	54.89	257.5	0.28	0.001	0.006
KARE Soil vs KARE Air	35.01	180.7	0.23	0.001	0.006
Hwy33 Soil vs Hwy33 Air	26.04	95.3	0.19	0.001	0.006
Hwy33 Soil vs KARE Air	24.38	96.1	0.23	0.001	0.006
Hwy33 Air vs KARE Air	5.89	21.6	0.08	0.001	0.006

Table S6. PERMANOVA coefficient table (*using community data rarefied to the mean sequencing depth*) for the Bray-Curtis dissimilarity among samples as a function of land use, site, year, month and sampling medium and the interactions between them in a fully nested model (adonis2 function). Permutations = 1000 (unstratified). n = 1002. df = degrees of freedom. F = pseudo F-ratio (Anderson, 2001). Note: very low p-values are likely a result of greatly increased sensitivity due to high replication (van der Laan et al., 2010), whereas r^2 and F values can better differentiate between important and trivial independent variables.

Model
~ Land Use + Site + Year + Month + Medium + Medium*Land Use/Site/Year/Month

	df	Sum of Squares	r^2	F	p value
Land Use	1	65.93	0.19	404.59	0.001
Site	4	7.57	0.02	11.61	0.001
Year	1	11.81	0.03	72.45	0.001
Month	11	17.91	0.05	9.99	0.001
Medium	1	37.06	0.11	227.41	0.001
Land Use : Medium	1	15.61	0.05	95.8	0.001
Land Use : Site : Medium	4	4.45	0.01	6.82	0.001
Land Use : Site : Year : Medium	10	4.98	0.01	3.05	0.001
Land Use : Site : Year : Month : Medium	104	40.37	0.12	2.38	0.001
Residual	864	140.8	0.41		
Total	1001	346.47	1		

Table S7. PERMANOVA coefficient table (*including sample sequencing depth as a predictor variable*) for the Bray-Curtis dissimilarity among samples as a function of land use, site, year, month and sampling medium and the interactions between them in a fully nested model (adonis2 function). Permutations = 1000 (unstratified). n = 1002. df = degrees of freedom. F = pseudo F-ratio (Anderson, 2001). Note: very low p-values are likely a result of greatly increased sensitivity due to high replication (van der Laan et al., 2010), whereas r^2 and F values can better differentiate between important and trivial independent variables.

Model
~ Land Use + Site + Year + Month + Medium + Sequencing Depth + Medium*Land Use/Site/Year/Month

	df	Sum of Squares	r^2	F	p value
Land Use	1	64.74	0.18	384.67	0.001
Site	4	7.73	0.02	11.48	0.001
Year	1	12.08	0.03	71.79	0.001
Month	11	17.6	0.05	9.51	0.001
Medium	1	35.95	0.1	213.59	0.001
Sequencing Depth	1	2.92	0.01	17.38	0.001
Land Use : Medium	1	14.88	0.04	88.43	0.001
Land Use : Site : Medium	4	4.62	0.01	6.87	0.001
Land Use : Site : Year : Medium	10	5.31	0.02	3.16	0.001
Land Use : Site : Year : Month : Medium	104	40.69	0.12	2.32	0.001
Residual	863	145.25	0.41		
Total	1001	351.78	1		

References

Abrego, N., Crosier, B., Somervuo, P., Ivanova, N., Abrahamyan, A., Abdi, A., Hämäläinen, K., Junninen, K., Maunula, M., & Purhonen, J. (2020). Fungal communities decline with urbanization—More in air than in soil. *The ISME Journal*, *14*(11), 2806–2815.

Abrego, N., Norros, V., Halme, P., Somervuo, P., Ali-Kovero, H., & Ovaskainen, O. (2018). Give me a sample of air and I will tell which species are found from your region: Molecular identification of fungi from airborne spore samples. *Molecular Ecology Resources*, *18*(3), 511–524.

Adams, R. I., Miletto, M., Taylor, J. W., & Bruns, T. D. (2013). Dispersal in microbes: Fungi in indoor air are dominated by outdoor air and show dispersal limitation at short distances. *The ISME Journal*, *7*(7), 1262–1273.

Anderson, M. J. (2001). A new method for non-parametric multivariate analysis of variance. *Austral Ecology*, *26*(1), 32–46.

Barberán, A., Ladau, J., Leff, J. W., Pollard, K. S., Menninger, H. L., Dunn, R. R., & Fierer, N. (2015). Continental-scale distributions of dust-associated bacteria and fungi. *Proceedings of the National Academy of Sciences*, *112*(18), 5756–5761.

Bowers, R. M., Lauber, C. L., Wiedinmyer, C., Hamady, M., Hallar, A. G., Fall, R., Knight, R., & Fierer, N. (2009). Characterization of airborne microbial communities at a high-elevation site and their potential to act as atmospheric ice nuclei. *Applied and Environmental Microbiology*, *75*(15), 5121–5130.

Cáliz, J., Triadó-Margarit, X., Camarero, L., & Casamayor, E. O. (2018). A long-term survey unveils strong seasonal patterns in the airborne microbiome coupled to general and

regional atmospheric circulations. *Proceedings of the National Academy of Sciences*, *115*(48), 12229–12234.

Castaño, C., Oliva, J., Martinez de Aragon, J., Alday, J. G., Parladé, J., Pera, J., & Bonet, J. A. (2017). Mushroom emergence detected by combining spore trapping with molecular techniques. *Applied and Environmental Microbiology*, *83*(13), e00600-17.

Chen, W., Hambleton, S., Seifert, K. A., Carisse, O., Diarra, M. S., Peters, R. D., Lowe, C., Chapados, J. T., & Lévesque, C. A. (2018). Assessing performance of spore samplers in monitoring aeromycobiota and fungal plant pathogen diversity in Canada. *Applied and Environmental Microbiology*, *84*(9), e02601-17.

Du, P., Du, R., Ren, W., Lu, Z., Zhang, Y., & Fu, P. (2018). Variations of bacteria and fungi in PM2.5 in Beijing, China. *Atmospheric Environment*, *172*, 55–64. https://doi.org/10.1016/j.atmosenv.2017.10.048

Fierer, N., Liu, Z., Rodríguez-Hernández, M., Knight, R., Henn, M., & Hernandez, M. T. (2008). Short-term temporal variability in airborne bacterial and fungal populations. *Applied and Environmental Microbiology*, *74*(1), 200–207.

Fröhlich-Nowoisky, J., Burrows, S., Xie, Z., Engling, G., Solomon, P., Fraser, M., Mayol-Bracero, O., Artaxo, P., Begerow, D., Conrad, R., & others. (2012). Biogeography in the air: Fungal diversity over land and oceans. *Biogeosciences*, *9*(3), 1125–1136.

Frohlich-Nowoisky, J., Pickersgill, D. A., Despres, V. R., & Poschl, U. (2009). High diversity of fungi in air particulate matter. *Proceedings of the National Academy of Sciences*, *106*(31), 12814–12819. https://doi.org/10.1073/pnas.0811003106

Kivlin, S. N., Winston, G. C., Goulden, M. L., & Treseder, K. K. (2014). Environmental filtering affects soil fungal community composition more than dispersal limitation at regional scales. *Fungal Ecology*, *12*, 14–25.

Nicolaisen, M., West, J. S., Sapkota, R., Canning, G. G., Schoen, C., & Justesen, A. F. (2017). Fungal communities including plant pathogens in near surface air are similar across northwestern Europe. *Frontiers in Microbiology*, *8*, 1729.

Niu, M., Hu, W., Cheng, B., Wu, L., Ren, L., Deng, J., Shen, F., & Fu, P. (2021). Influence of rainfall on fungal aerobiota in the urban atmosphere over Tianjin, China: A case study. *Atmospheric Environment: X*, *12*, 100137.

Núñez, A., & Moreno, D. A. (2020). The differential vertical distribution of the airborne biological particles reveals an atmospheric reservoir of microbial pathogens and aeroallergens. *Microbial Ecology*, *80*(2), 322–333.

Peay, K. G., & Bruns, T. D. (2014). Spore dispersal of basidiomycete fungi at the landscape scale is driven by stochastic and deterministic processes and generates variability in plant–fungal interactions. *New Phytologist*, *204*(1), 180–191.

Redondo, M. A., Berlin, A., Boberg, J., & Oliva, J. (2020). Vegetation type determines spore deposition within a forest–agricultural mosaic landscape. *FEMS Microbiology Ecology*, *96*(6), fiaa082.

Redondo, M. A., Oliva, J., Elfstrand, M., Boberg, J., Capador-Barreto, H. D., Karlsson, B., & Berlin, A. (2022). Host genotype interacts with aerial spore communities and influences the needle mycobiome of Norway spruce. *Environmental Microbiology*.

Sánchez-Parra, B., Núñez, A., García, A. M., Campoy, P., & Moreno, D. A. (2021). Distribution of airborne pollen, fungi and bacteria at four altitudes using high-throughput DNA sequencing. *Atmospheric Research*, *249*, 105306.

Schiro, G., Chen, Y., Blankinship, J. C., & Barberán, A. (2022). Ride the dust: Linking dust dispersal and spatial distribution of microorganisms across an arid landscape (Accepted). *Environmental Microbiology*. https://doi.org/10.1111/1462-2920.15998

Tignat-Perrier, R., Dommergue, A., Thollot, A., Keuschnig, C., Magand, O., Vogel, T. M., & Larose, C. (2019). Global airborne microbial communities controlled by surrounding landscapes and wind conditions. *Scientific Reports*, *9*(1), 1–11.

Tipton, L., Zahn, G., Datlof, E., Kivlin, S. N., Sheridan, P., Amend, A. S., & Hynson, N. A. (2019). Fungal aerobiota are not affected by time nor environment over a 13-y time series at the Mauna Loa Observatory. *Proceedings of the National Academy of Sciences*, *116*(51), 25728–25733.

van der Laan, M., Hsu, J.-P., Peace, K. E., & Rose, S. (2010). Statistics ready for a revolution: Next generation of statisticians must build tools for massive data sets. *AMSTAT News: The Membership Magazine of the American Statistical Association*, *399*, 38–39.

Womack, A. M., Artaxo, P., Ishida, F. Y., Mueller, R. C., Saleska, S. R., Wiedemann, K. T., Bohannan, B. J., & Green, J. L. (2015). Characterization of active and total fungal communities in the atmosphere over the Amazon rainforest. *Biogeosciences*, *12*(21), 6337–6349.

Woo, C., An, C., Xu, S., Yi, S.-M., & Yamamoto, N. (2018). Taxonomic diversity of fungi deposited from the atmosphere. *The ISME Journal*, *12*(8), 2051–2060. https://doi.org/10.1038/s41396-018-0160-7

Yamamoto, N., Bibby, K., Qian, J., Hospodsky, D., Rismani-Yazdi, H., Nazaroff, W. W., & Peccia, J. (2012). Particle-size distributions and seasonal diversity of allergenic and pathogenic fungi in outdoor air. *The ISME Journal*, *6*(10), 1801–1811.

Yan, D., Zhang, T., Su, J., Zhao, L.-L., Wang, H., Fang, X.-M., Zhang, Y.-Q., Liu, H.-Y., & Yu, L.-Y. (2016). Diversity and composition of airborne fungal community associated with particulate matters in Beijing during haze and non-haze days. *Frontiers in Microbiology*, *7*, 487.
